# Supplementary figures and images for: LRRC15 mediates an accessory interaction with the SARS-CoV-2 spike protein
Source: PLoS Biol. 2023 Feb 3;21(2):e3001959. doi: 10.1371/journal.pbio.3001959 (PMC9897555; doi:10.1371/journal.pbio.3001959)

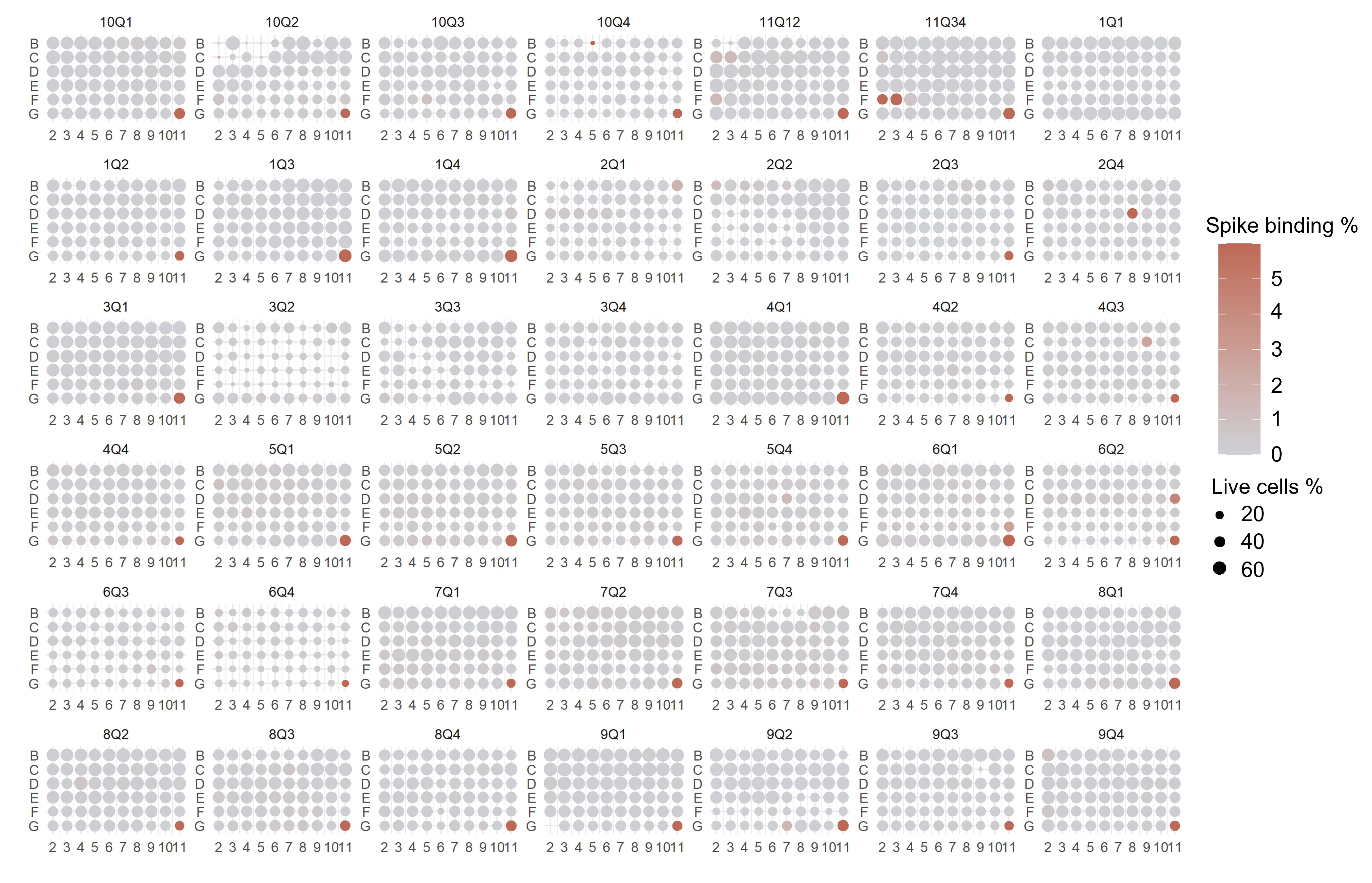

Supplement: S1 Fig — Each well measured by flow cytometry contains cells transfected with an expression plasmid encoding full-length cDNA from our library encompassing the vast majority of human cell surface receptors. A majority of measured plates included a positive control well of ACE2 in the bottom right corner. As shown in main Fig 1C, hits from this screen aside from LRRC15 and already known spike receptors were found to be artifacts upon replication. ACE2, angiotensin-converting enzyme 2; LRRC15, leucine-rich repeat containing protein 15. (TIF) [file pbio.3001959.s002.tif]

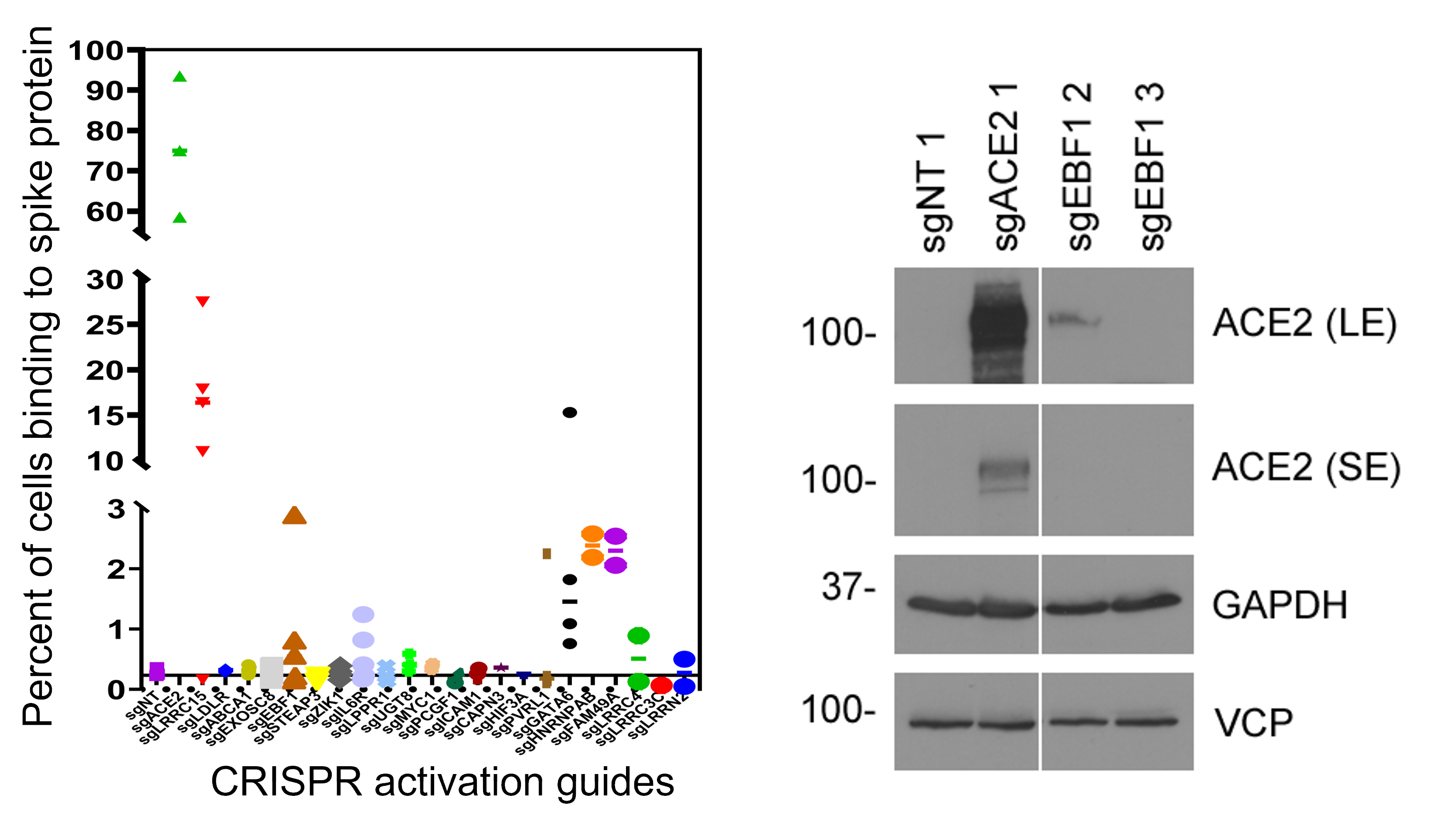

Supplement: S2 Fig — An overview of binding signals upon replicating each sgRNA is shown (left) alongside western blots that validate one EBF1 sgRNA as slightly up-regulating ACE2 expression in RPE1 cells. Two exposures are shown, long (LE) and short (SE). The nearest molecular mass (in kilodaltons) of the standard is indicated next to each blot. ACE2, angiotensin-converting enzyme 2; CRISPRa, CRISPR activation. (TIF) [file pbio.3001959.s003.tif]

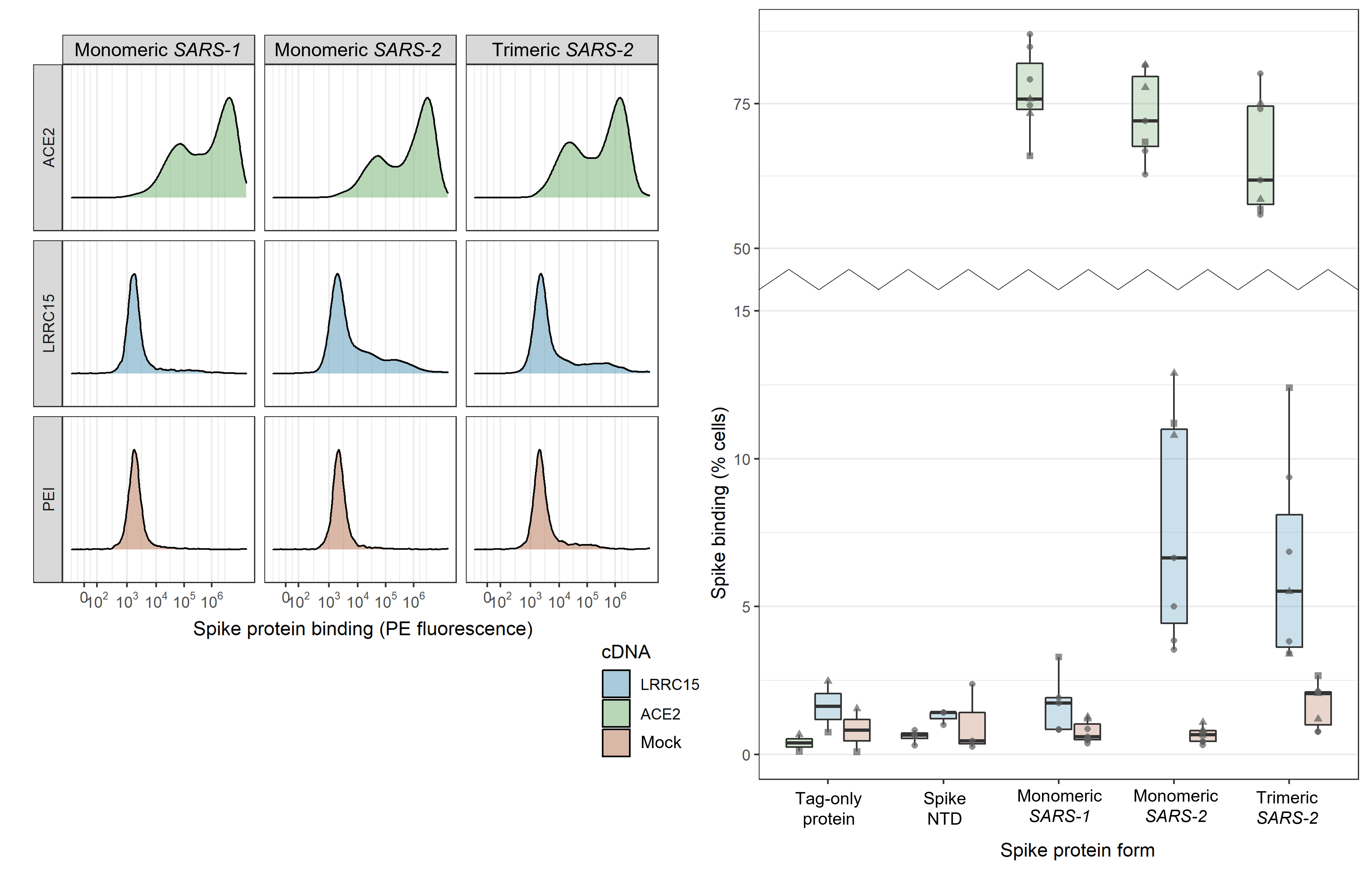

Supplement: S3 Fig — Representative flow cytometry traces depicting recombinant spike protein binding to HEK293 cells transfected to express the indicated receptors (left) are shown next to the quantified percentages of cells that bound spike. The y-axis is truncated at the indicated break point in order to display all conditions in a single plot. LRRC15, leucine-rich repeat containing protein 15; SARS-CoV-2, Severe Acute Respiratory Syndrome Coronavirus 2. (TIF) [file pbio.3001959.s004.tif]

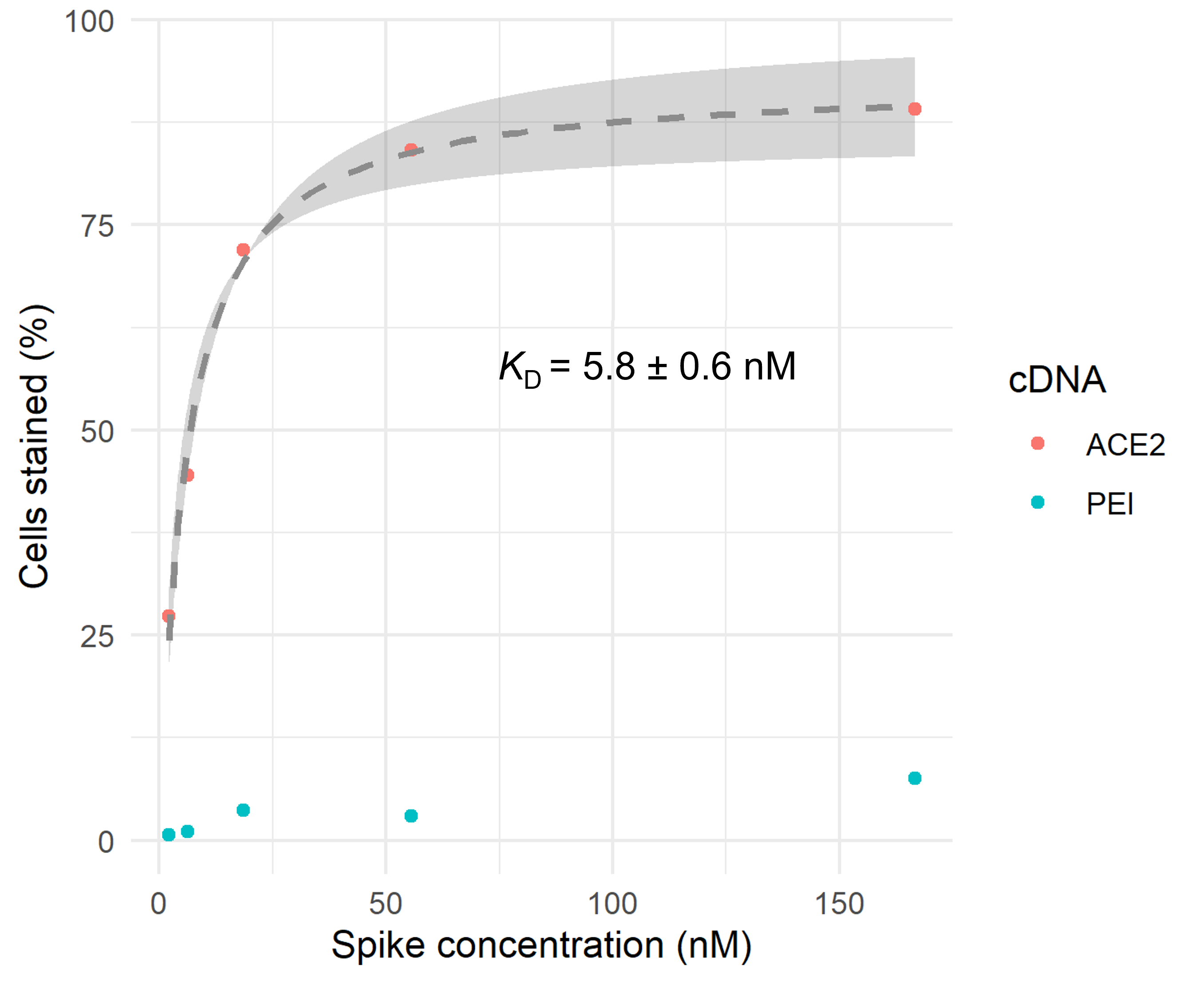

Supplement: S4 Fig — After incubating ACE2-transfected HEK293 cells with a range of monomeric spike protein concentrations, the binding saturation curve was measured to estimate an equilibrium dissociation constant. ACE2, angiotensin-converting enzyme 2. (TIF) [file pbio.3001959.s005.tif]

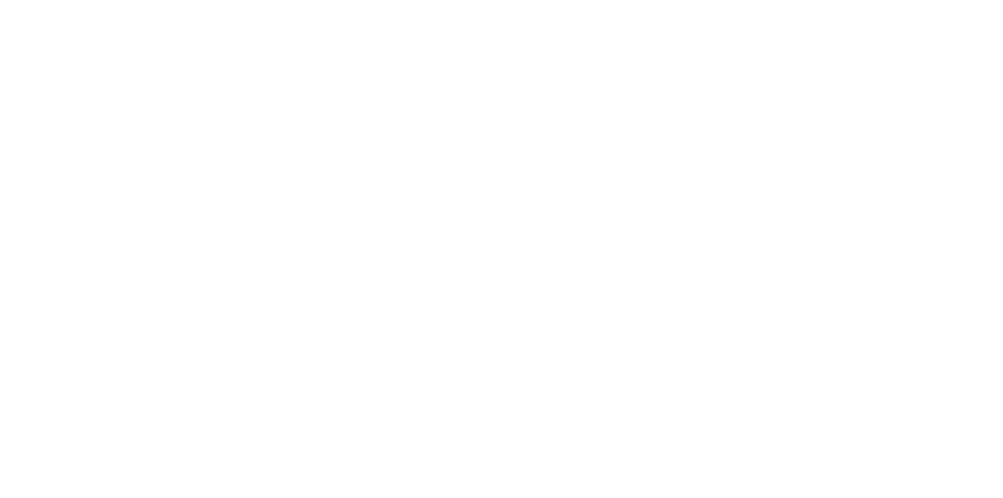

Supplement: S5 Fig — qPCR measurements of mRNA for ACE2 or LRRC15 in cell lines transduced for the sgRNAs indicated along the x-axis. ACE2, angiotensin-converting enzyme 2; CRISPRa, CRISPR activation; LRRC15, leucine-rich repeat containing protein 15. (TIF) [file pbio.3001959.s006.tif]

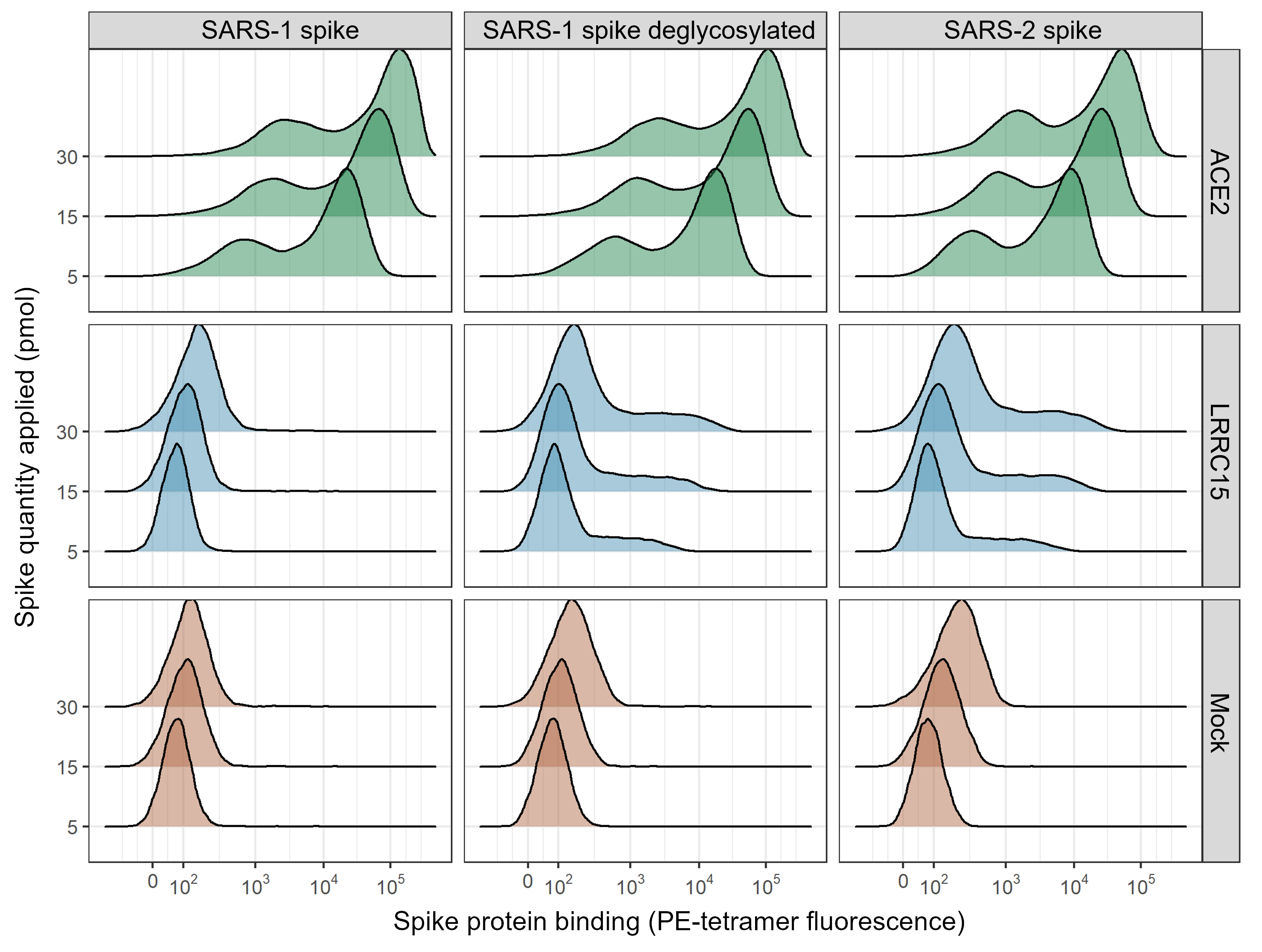

Supplement: S6 Fig — Comparison of SARS-CoV-1 spike to SARS-CoV-2 spike, where enzymatic removal of most N-linked glycans by PNGase F results in the SARS-CoV-1 spike gaining the ability to bind LRRC15 at similar levels to SARS-CoV-2 spike. LRRC15, leucine-rich repeat containing protein 15; SARS-CoV-1, Severe Acute Respiratory Syndrome Coronavirus 1; SARS-CoV-2, Severe Acute Respiratory Syndrome Coronavirus 2. (TIF) [file pbio.3001959.s007.tif]

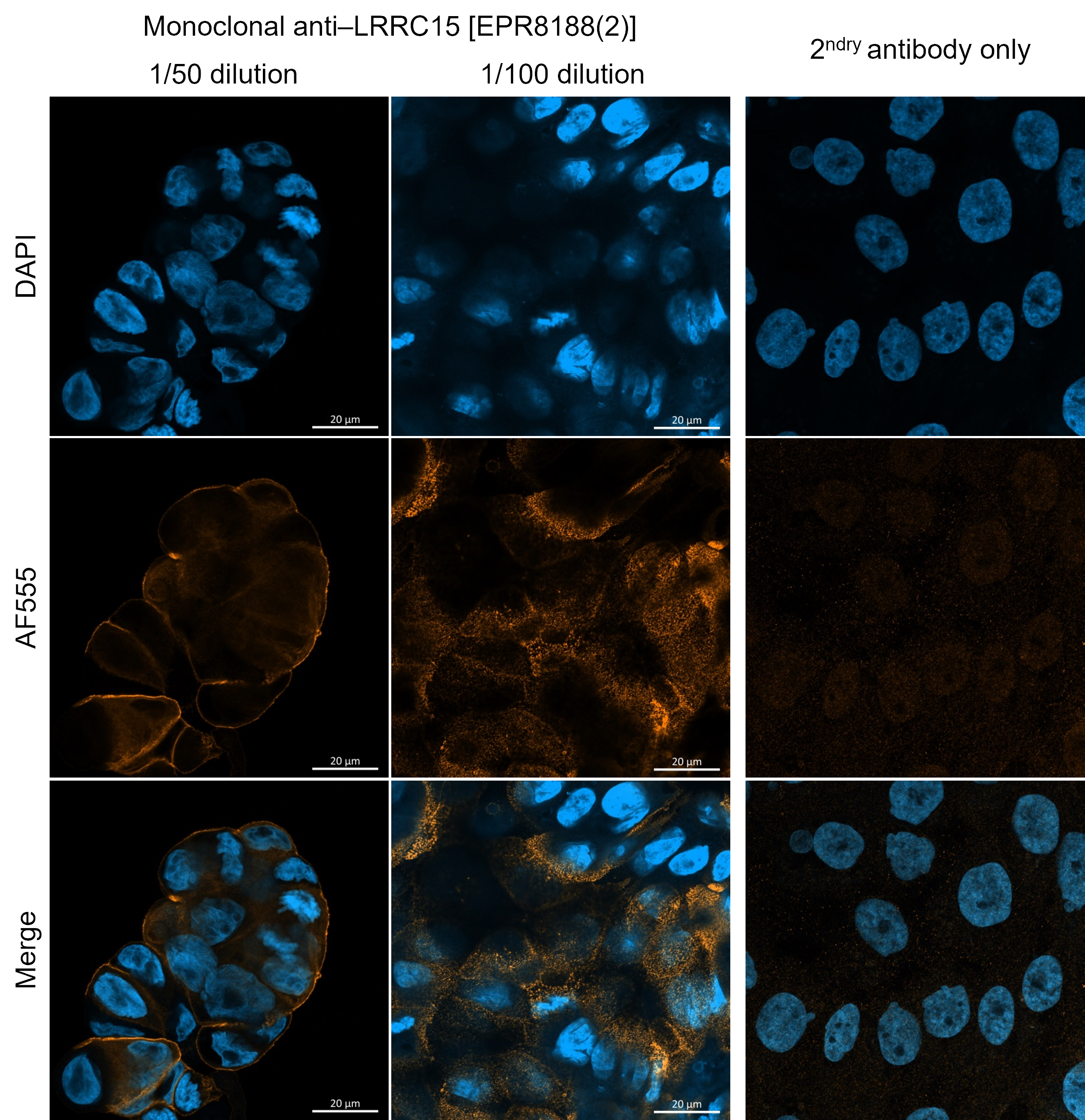

Supplement: S7 Fig — Two fields of view are shown for CaLu-3 cells stained at different dilutions of monoclonal anti- human LRRC15 antibody. LRRC15 was predominantly detected along the cell plasma membrane, with some possible faint staining in intracellular compartments. LRRC15, leucine-rich repeat containing protein 15. (TIF) [file pbio.3001959.s008.tif]

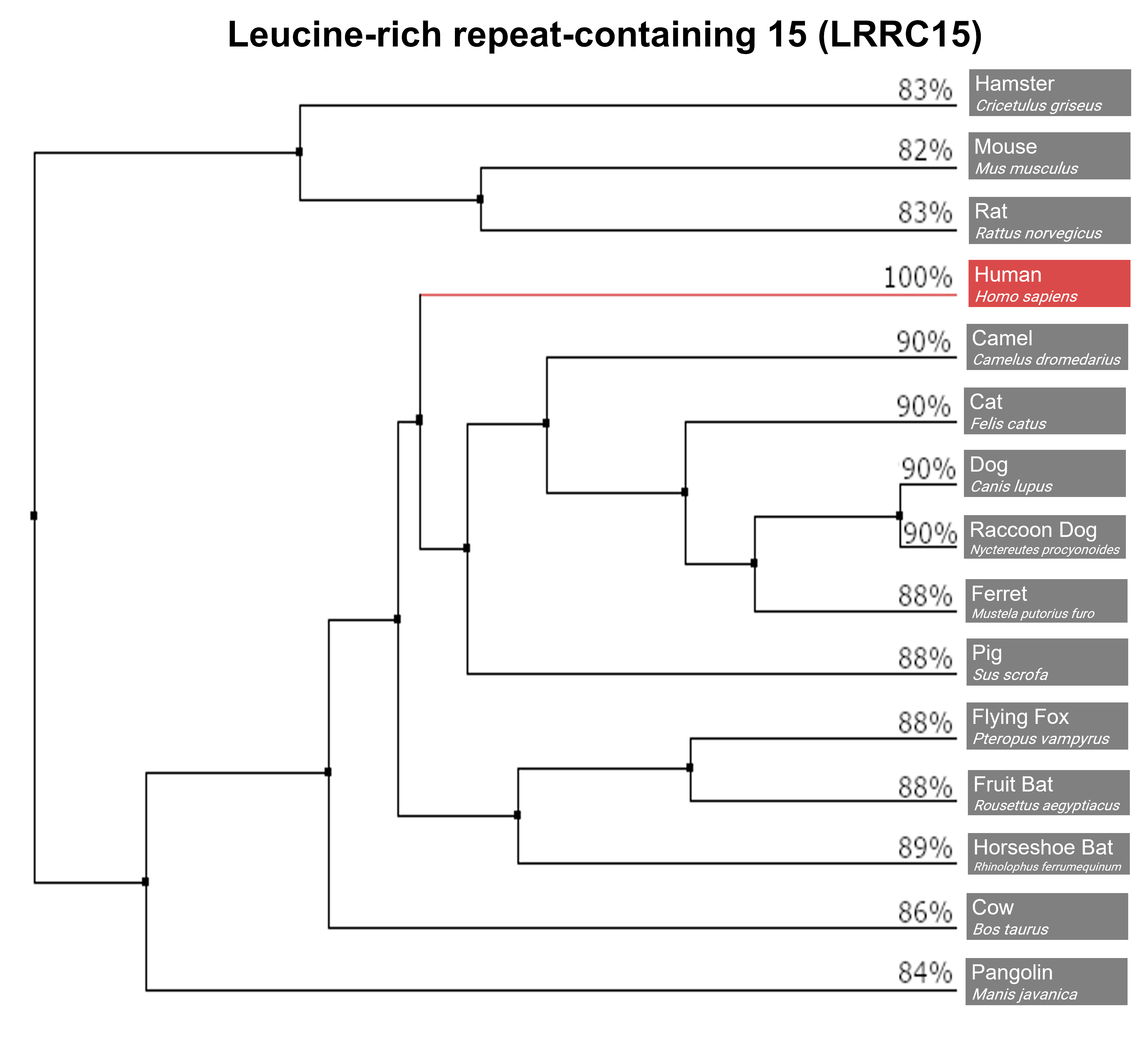

Supplement: S10 Fig — The percentages of LRRC15 residues that are identical to human are shown at each terminal branch. LRRC15, leucine-rich repeat containing protein 15; SARS-CoV-2, Severe Acute Respiratory Syndrome Coronavirus 2. (TIF) [file pbio.3001959.s011.tif]

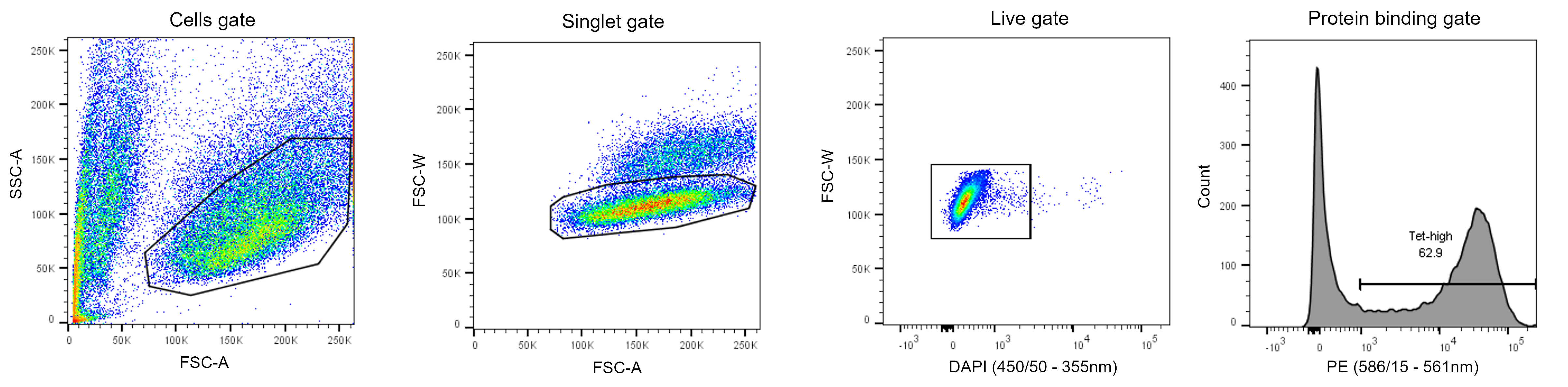

Supplement: S11 Fig — The hierarchy of gates goes from left to right. (TIF) [file pbio.3001959.s012.tif]
