# Supplementary material for: LRRC15 mediates an accessory interaction with the SARS-CoV-2 spike protein
Source: PLoS Biol. 2023 Feb 3;21(2):e3001959. doi: 10.1371/journal.pbio.3001959 (PMC9897555; doi:10.1371/journal.pbio.3001959)

Original blot
Supplementary Figure 2

Blot used in figure

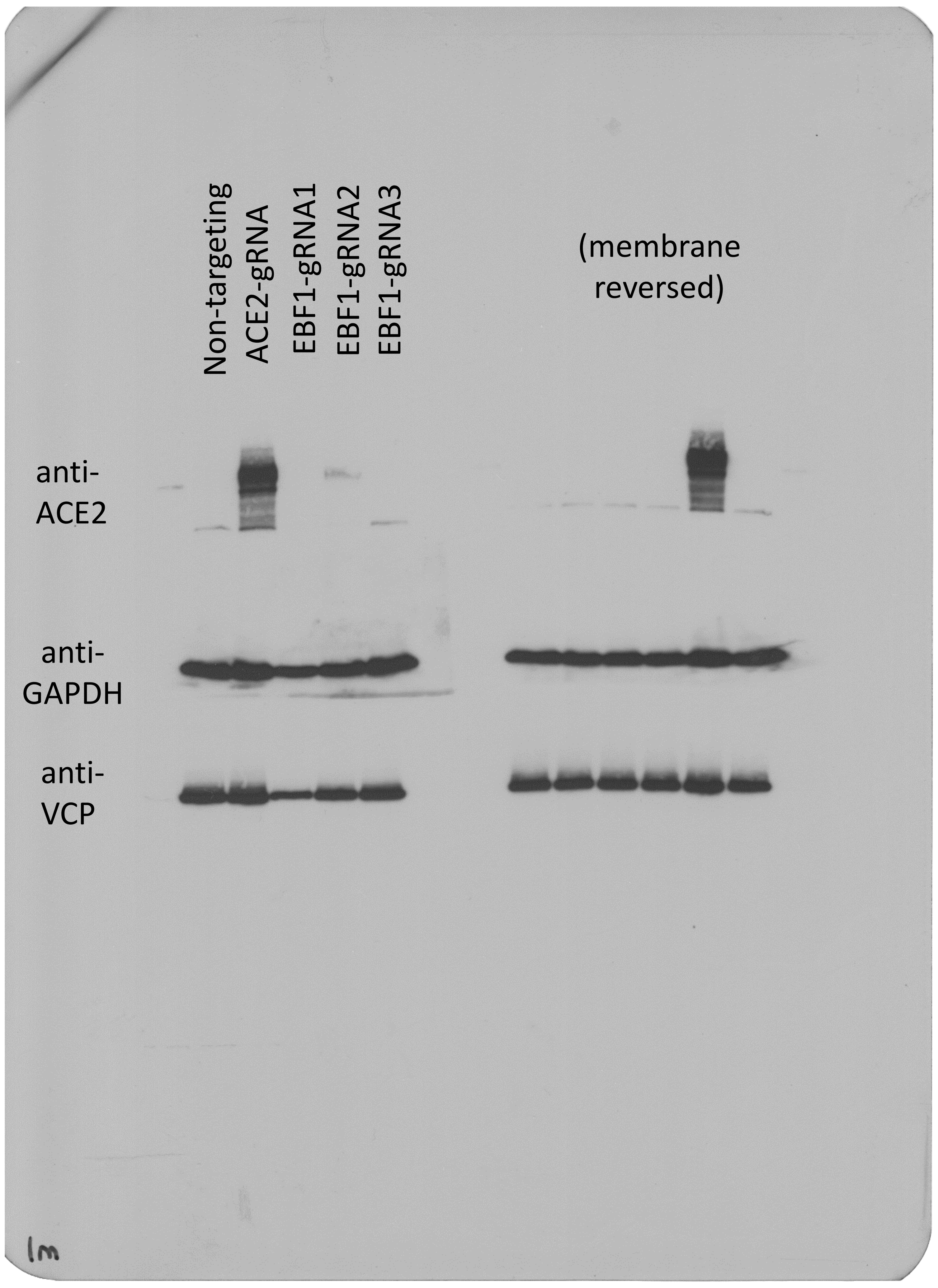

Molecular weight marker and original annotations

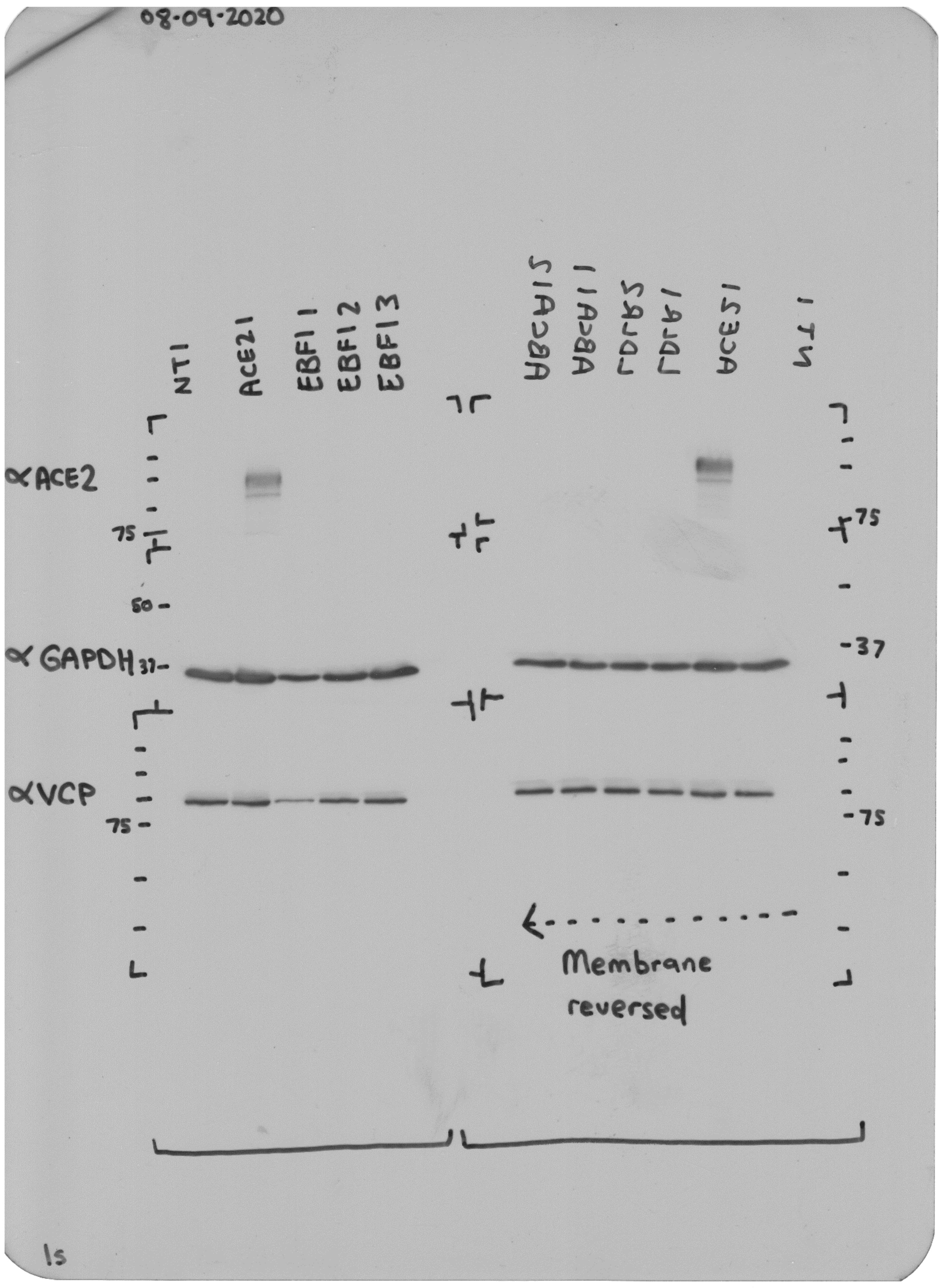

Original blot
Figure 2 panel F, left side

LRRC15 exposure blot

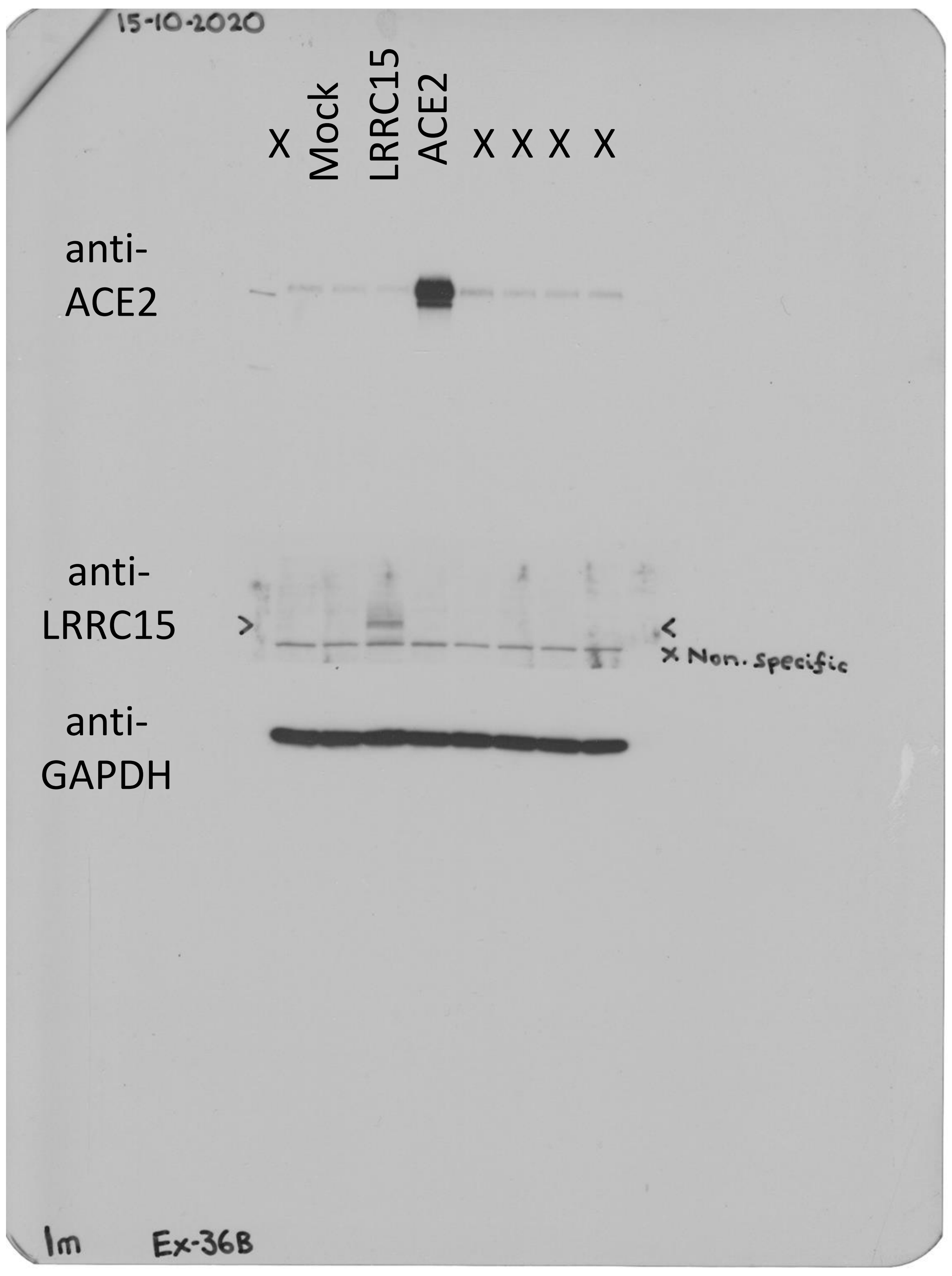

ACE2 long exposure blot

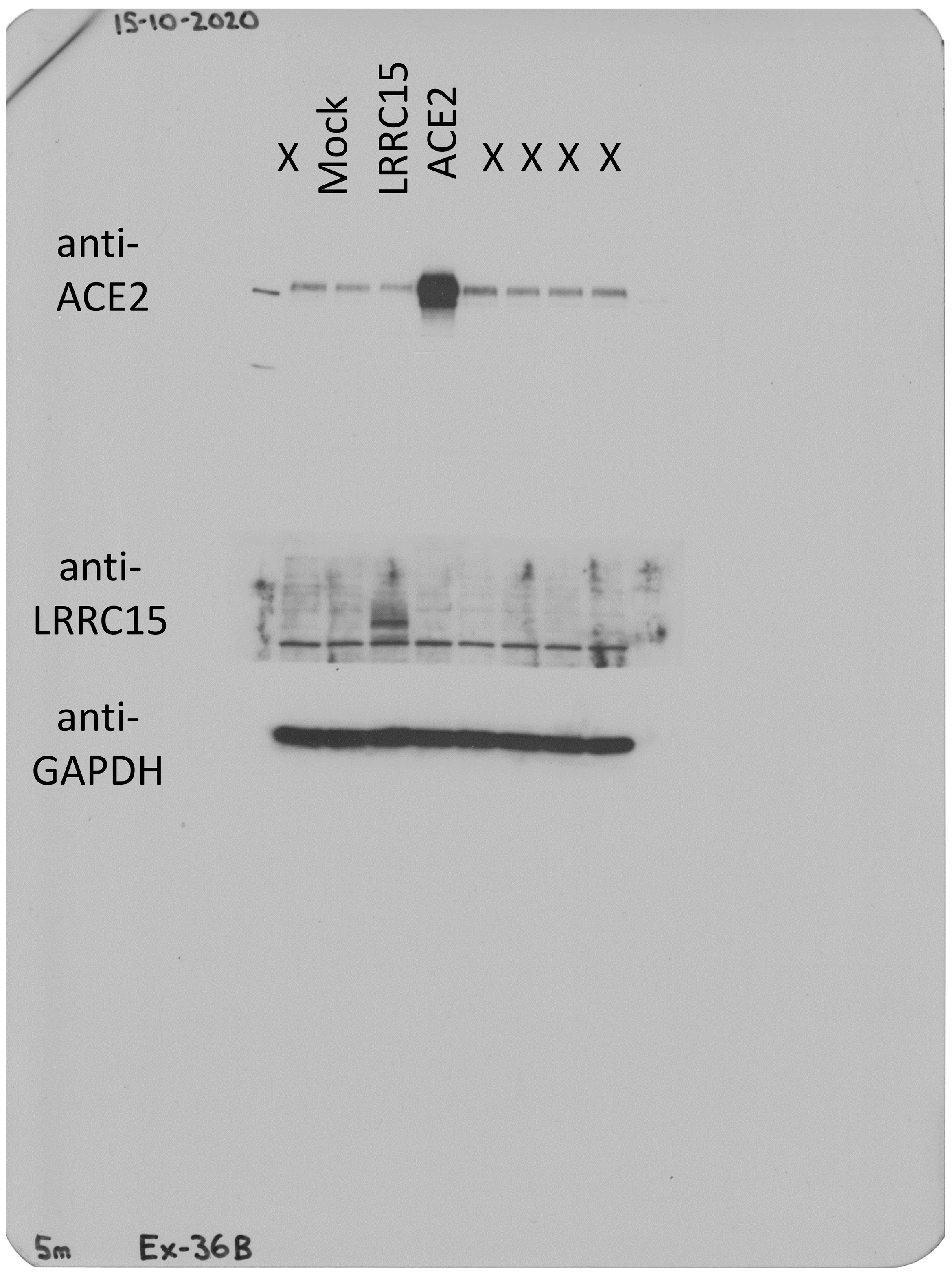

Molecular weight marker and ACE2 short exposure

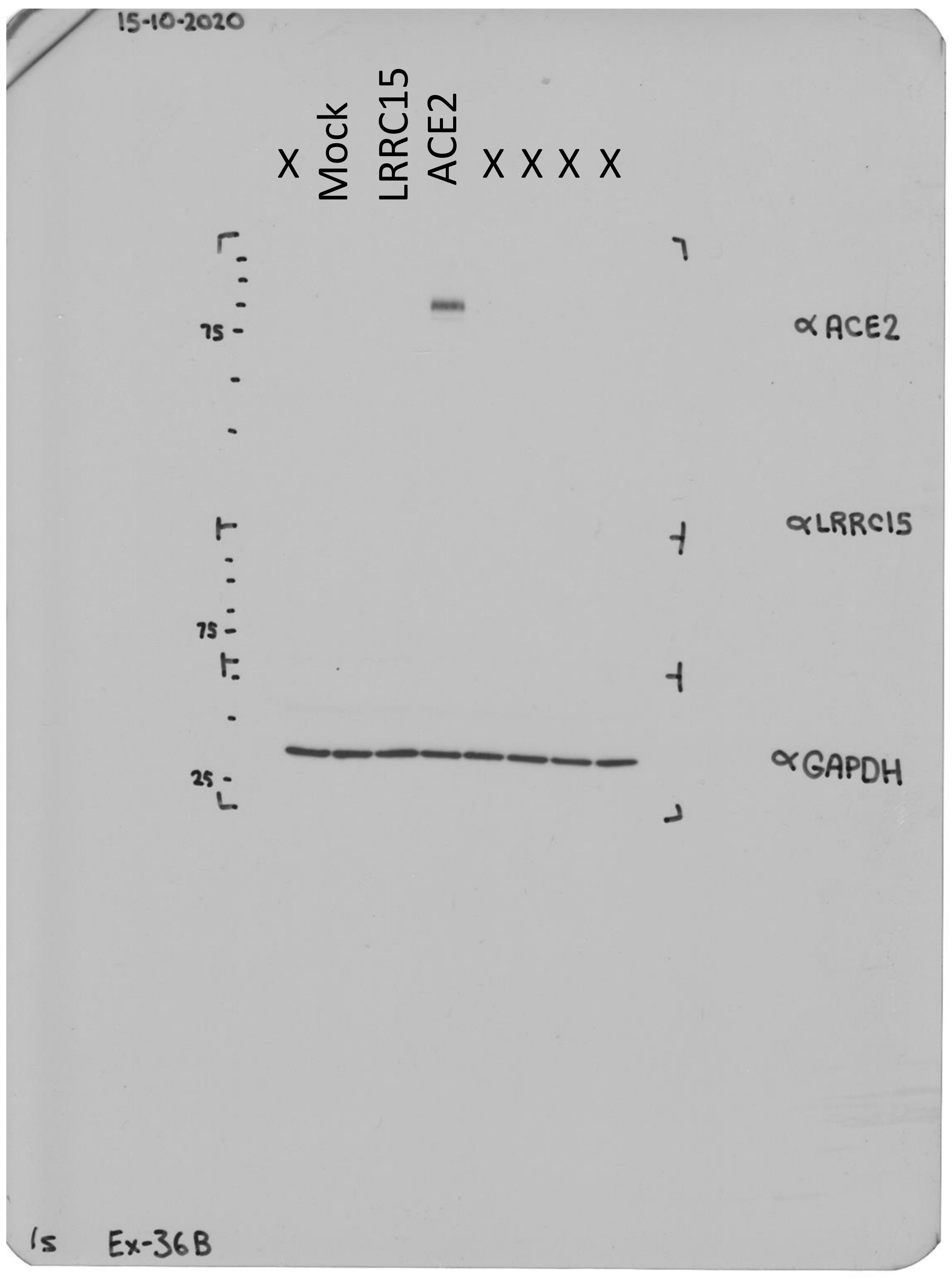

Original blot
Figure 2 panel F, right side

ACE2 short exposure blot

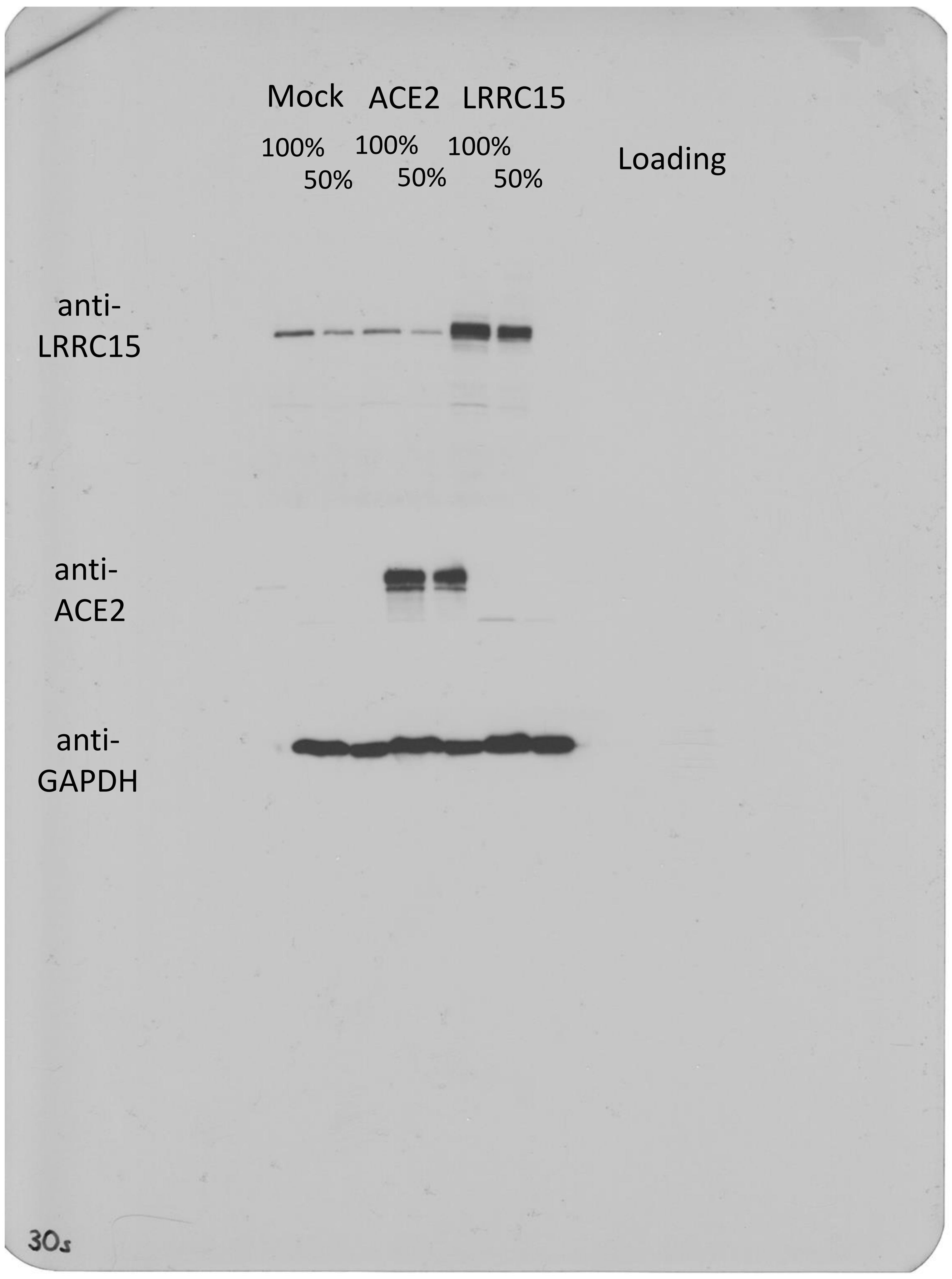

ACE2 long exposure blot

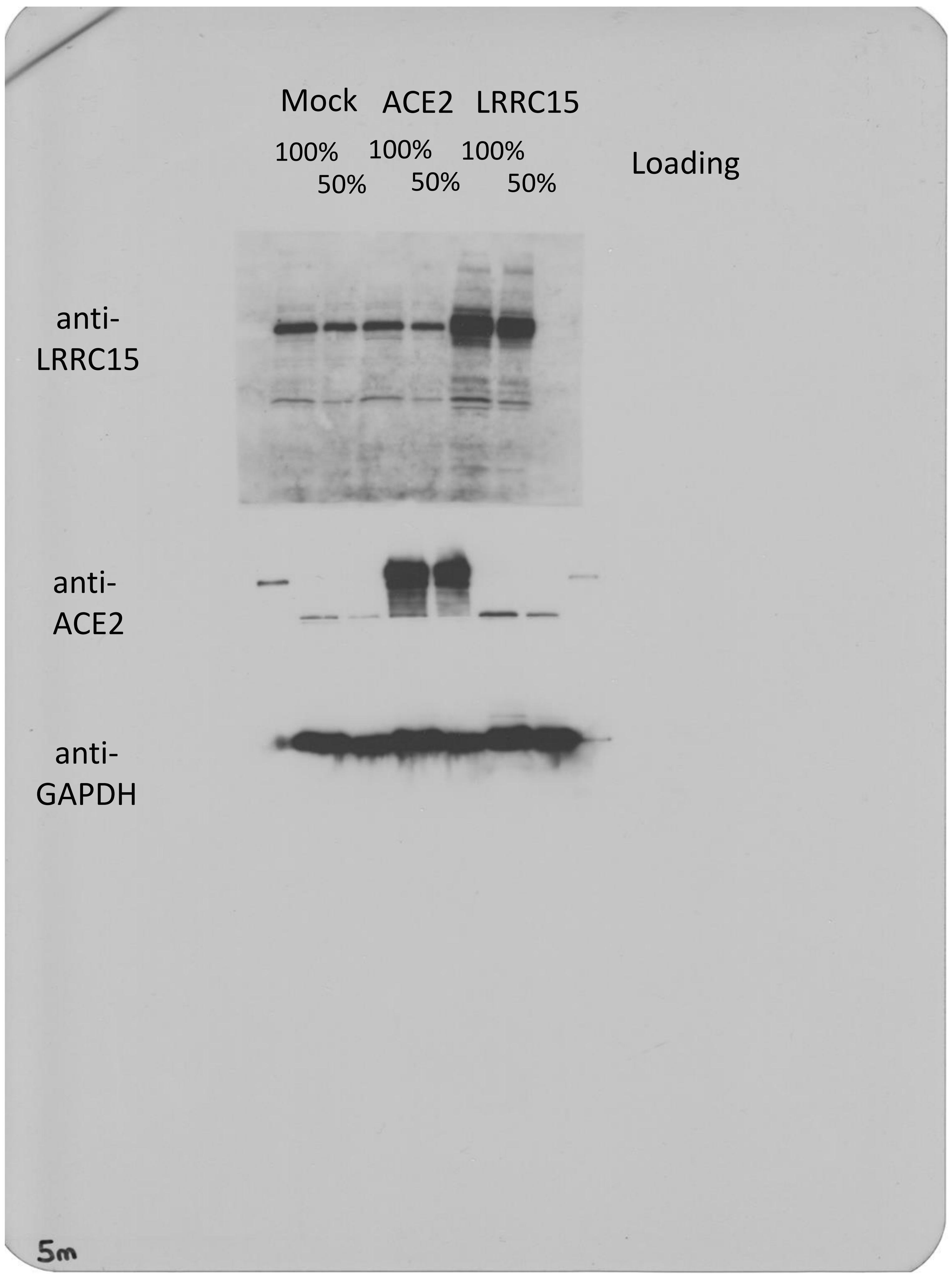

GAPDH exposure blot

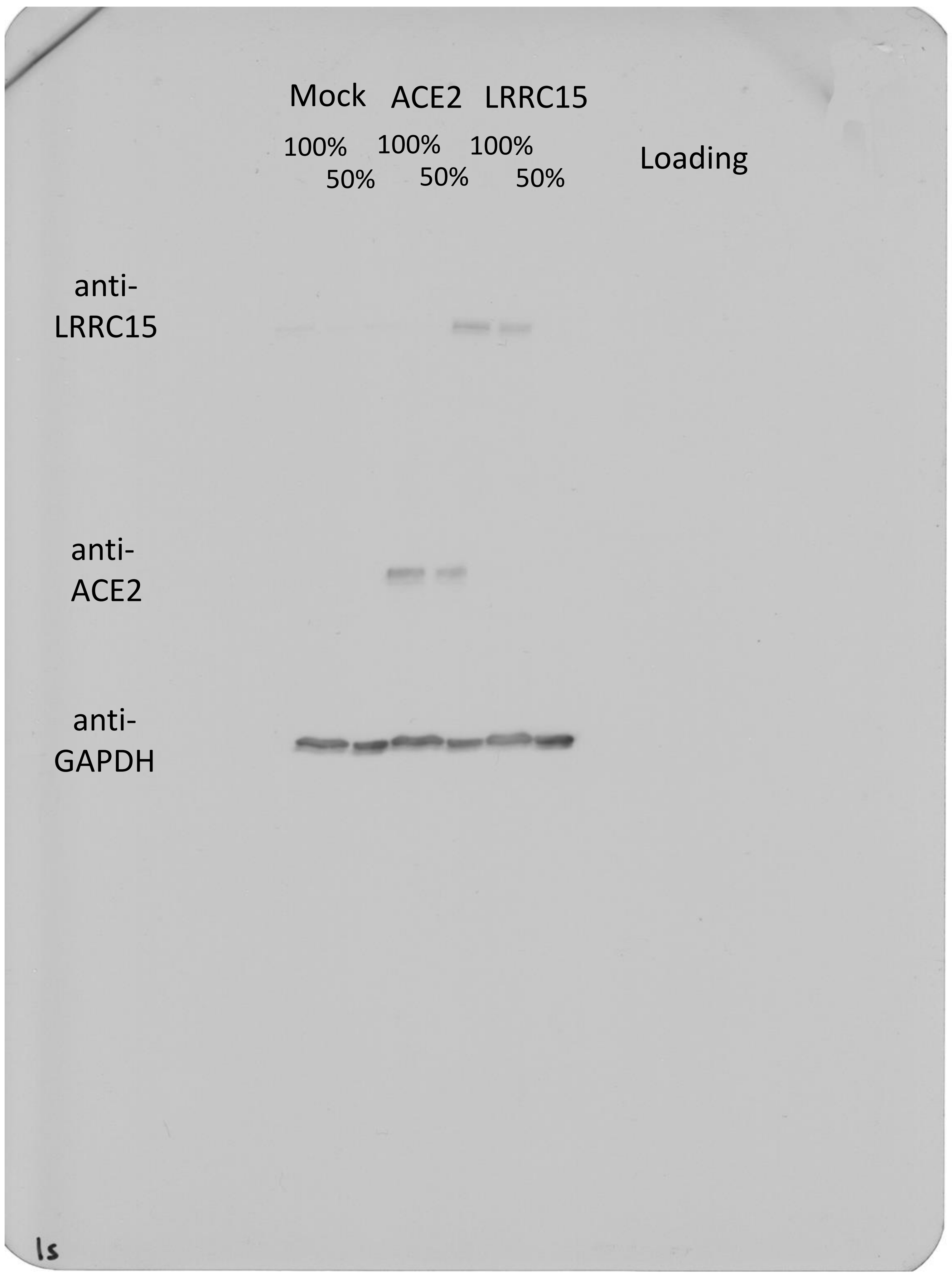

Molecular weight marker
and original annotations

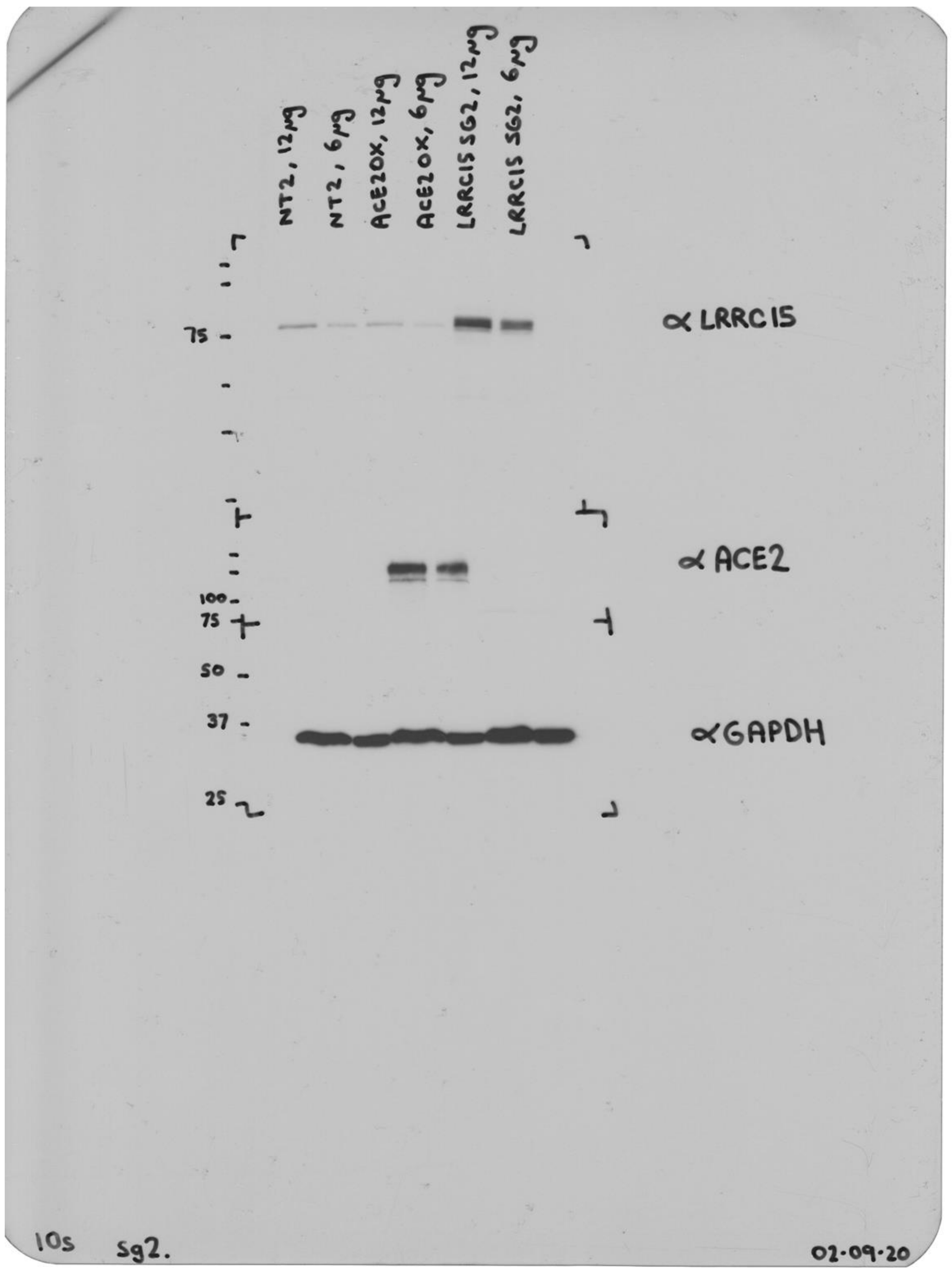

Original gels

Figure 3 panel A

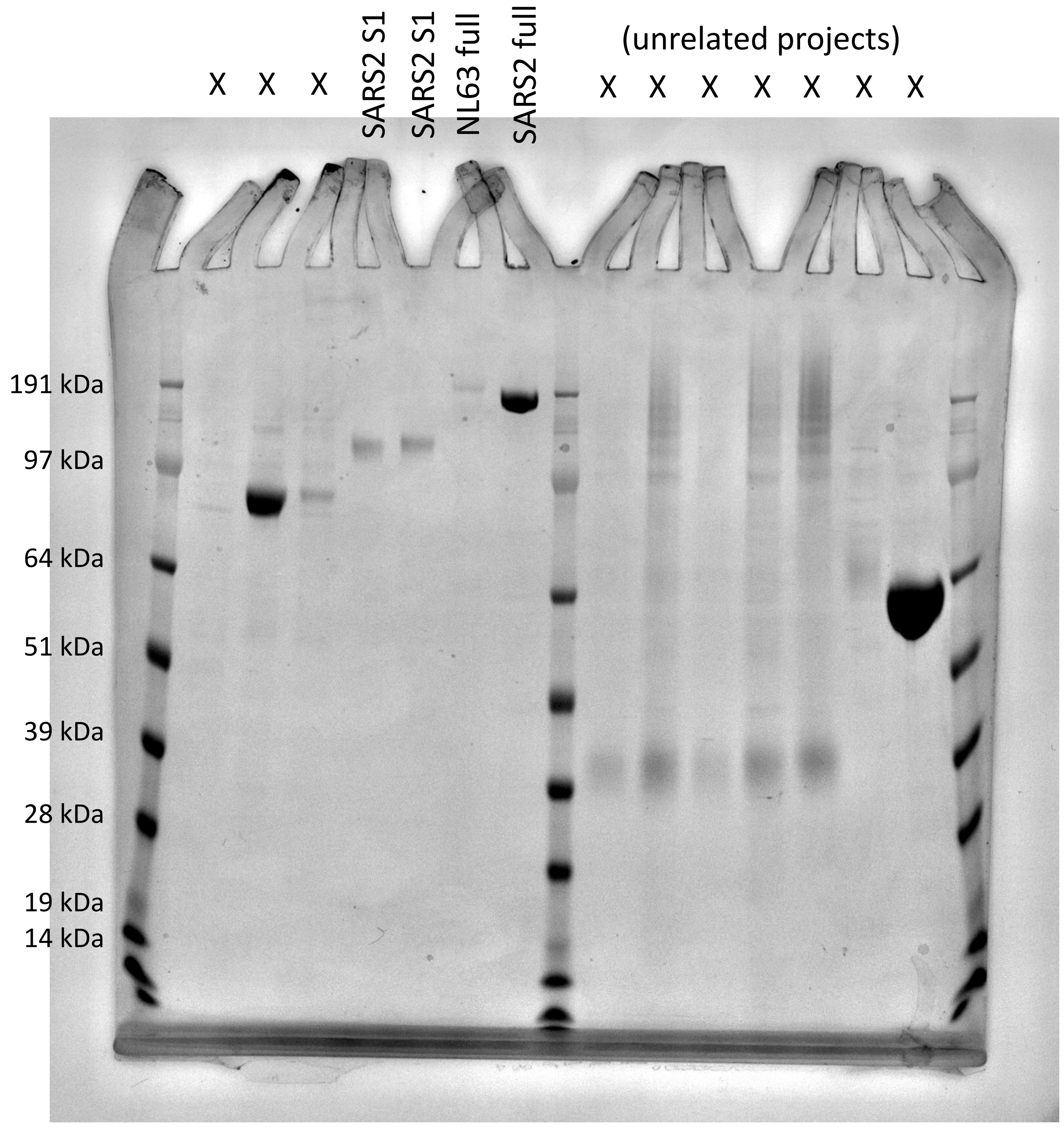

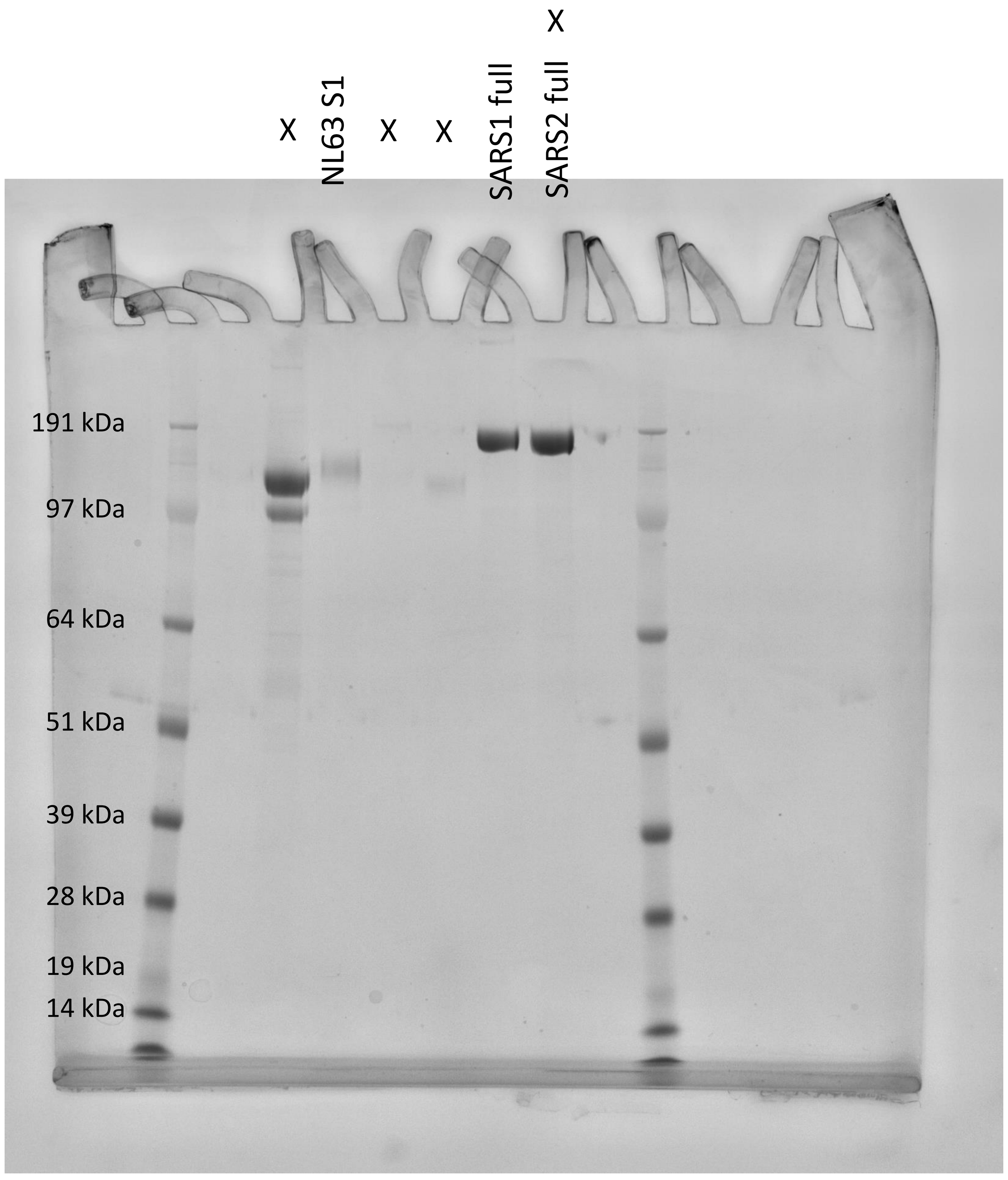

Supplement: S1 Raw Images — (PDF) [file pbio.3001959.s044.pdf]
