# Supplementary material for: LRRC15 mediates an accessory interaction with the SARS-CoV-2 spike protein
Source: PLoS Biol. 2023 Feb 3;21(2):e3001959. doi: 10.1371/journal.pbio.3001959 (PMC9897555; doi:10.1371/journal.pbio.3001959)
Supplement: Attachment — Submitted filename: LRRC15 SARScov2 rebuttal document REVISED1.pdf [file pbio.3001959.s045.pdf]

PBIOLOGY-D-21-02640R1

Thank you for submitting your manuscript "LRRC15 mediates an accessory interaction with the SARS-CoV-2 spike protein" for consideration as a Short Reports at PLOS Biology. Your manuscript has been evaluated by the PLOS Biology editors, an Academic Editor with relevant expertise, and by several independent reviewers.

In light of the reviews (below), we will not be able to accept the current version of the manuscript, but we would welcome re-submission of a much-revised version that takes into account the reviewers' comments. We cannot make any decision about publication until we have seen the revised manuscript and your response to the reviewers' comments. Your revised manuscript is also likely to be sent for further evaluation by the reviewers.

You will see that all the reviewers agree and have similar concerns. They all find the work novel and interesting but they all have issues that should be solved before publication. In particular, you should use the whole virus, test LRRC15 and ACE2 co-expression, and provide more rigorous data about enhancement of viral infection.

We would like to thank the reviewers again for their very constructive input on our study reporting a novel interaction between the SARS-CoV-2 spike protein and human LRRC15. In our revised manuscript, we have made several significant updates to reinforce our conclusions including (as a non-exhaustive summary) :

1) new analysis of data from COVID-19 patient lungs showing substantial upregulation of LRRC15 (as well as an increased distribution of ACE2), which both broadens the co-localization between ACE2 and LRRC15 in disease settings and raises the plausibility that levels of LRRC15 are sufficient to have a functionally-significant role. These analyses have been added to our revised Figure 4.

2) extensive additional binding data to reinforce and extend our earlier conclusions, including localizing the binding epitope of the spike to the RBD, verifying the binding of spike preparations that more closely resemble the spike conformations seen on virions, tests of other claimed spike receptors not found by our screening, and careful attempts at recombinant protein binding assays. These data have been added to our revised Figure 3, and as a new supplemental figure.

3) additional data and discussion on the roles LRRC15 could play in infection, including experimental tests of effects on infection in both *cis* and *trans*. We believe these data provide a foundation for future studies to build off the initial characterization we describe in this *Short Report*. In an exciting development, we also cite in our revised manuscript newly-collected clinical data from a recent preprint that independently found LRRC15 protein levels as having a strong predictive value for the prognosis of COVID-19 patients. The new data have been added as a supplemental figure, and both the final results section and related discussion paragraphs have been revised.

Lastly, we were delighted to see that in the months following when we submitted our manuscript for review (and concurrently posted a preprint to BioRxiv sharing our work), there have been two

separate research groups who subsequently posted their own preprints replicating all of our core findings about the interaction between LRRC15 and the SARS-CoV-2 spike protein (Song *et al*, Loo *et al*). Where appropriate, we now reference those studies for the compelling additional supporting evidence they provide, including where these studies address questions posed by the reviewers. We also cite these preprints when discussing the functional importance of LRRC15 in infection, based on both direct clinical associations and infection experiments described by those subsequent studies.

We believe this remarkable convergence of results following from our initial discovery concretely demonstrates the value our own study provides as a foundation for this emerging and fascinating new area of coronavirus research.

Reviewer #1: Teunis B.H. Geijtenbeek. Lectin type and other virus receptors.
Reviewer #1:
This study has identified LRRC15 as a protein that interacts with the Spike protein from SARS-CoV-2. Two independent screens have been performed and identified LRRC15. Further studies showed that LRRC15 interacts at the CTD of S protein and overlapping with ACE2 binding site. The authors suggest that LRRC15 is expressed in the same tissues but overlap with ACE2 expression is not very strong when analysing singe cell transcriptome data sets from public domain. Expression of LRRC15 did not render cells susceptible to SARS_CoV-2 recombinant virus. The authors suggest that LRRC15 might enhance infection as ectopic expression of LRRC15 in Calu-3 cells increases infection at low concentrations.

Identification of LRRC15 as a protein that interacts with S protein from SARS-CoV-2 is interesting and novel. Binding data with soluble S proteins and overexpression LRRC15 are convincing, although binding is weak compared to ACE2. Using public datasets the authors suggest that there is some overlap with ACE2 in the lung but these data are not very convincing. Functional relevance to SARS-CoV-2 remains unclear; LRRC15 overexpression did not allow infection by a recombinant SARS-CoV-2 in absence of ACE2. Based on ectopic expression in Calu-3 cells the authors suggest that it might enhance infection but this conclusion is not fully supported by the infection data. Finally, as the authors have not shown any convincing co-expression of LRRC15 with ACE2, it remains unclear what the relevance is of ectopic expression of LRRC15. Could LRRC15 be induced by inflammation or stimuli?

We agree that some of the ambiguities around the relevance of LRRC15 levels in the lung would be strengthened by a further more detailed analysis of how LRRC15 levels might respond to inflammation and other stimuli that accompany COVID-19. First, we considered whether LRRC15 levels might be influenced by the presence of cytokines, as would be common in the inflammatory environment of lungs experiencing severe COVID-19. Based on our single-cell analysis of the distribution of *LRRC15* in healthy non-inflamed lungs, we began by investigating the lung fibroblasts that appeared to have the highest basal levels of *LRRC15*.

We searched the Gene Expression Omnibus (GEO) database for publicly available datasets comparing human lung fibroblasts exposed to inflammatory cytokines compared to untreated controls. We identified 3 studies that performed controlled stimulation experiments on normal human lung fibroblasts. Despite using slightly different cell models and cytokine conditions, all 3 studies observed a marked upregulation of *LRRC15* expression (Figure R1A). On average across the studies, human lung fibroblasts increased *LRRC15* transcription by a factor of 4-fold higher (aggregated one-sample t-test $t = 5.7$, $p = 2x10^{-5}$).

We verified that this same upregulation of *LRRC15* takes place within the lungs of COVID-19 patients by analyzing data from a recent single-cell RNA sequencing study that compared lung tissue taken from patients with fatal COVID-19 to lung tissue resected from healthy individuals. Consistent with our earlier findings that inflammation greatly increases LRRC15 levels, we observed a significant increase in COVID-19 lung fibroblasts compared to controls (Figure R1B, Mann-Whitney AUC = 0.74, p = 0.066) (with the exception of one outlier point of a control patient with more than 10-fold greater *LRRC15* levels than any other control. Detailed metadata were not available for this patient to clarify if the medical reason why a lung resection biopsy was performed on them might account for this difference).

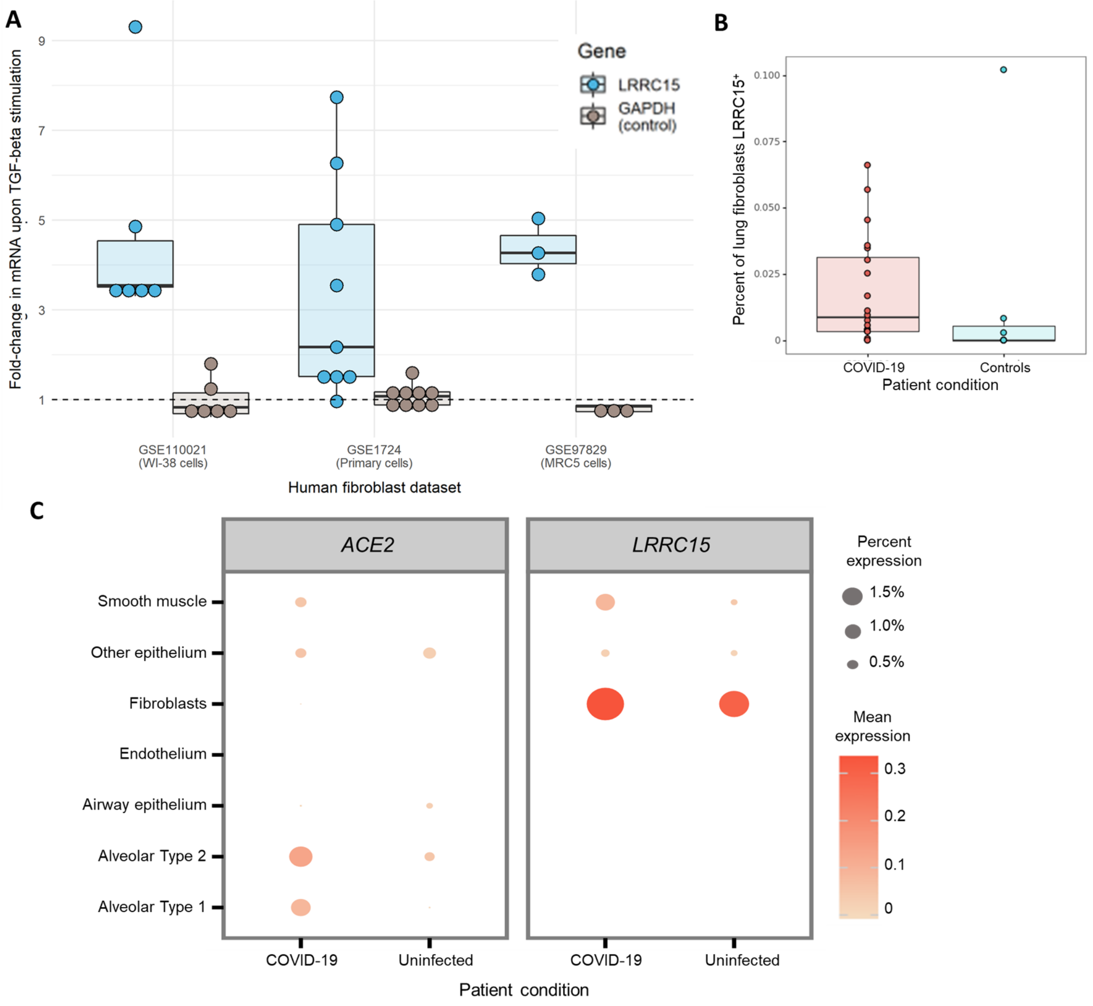

Figure R1 (*added to the revised manuscript in main figure 4*). Inflammation associated with COVID-19 dramatically increases the expression levels of *LRRC15* in lung cell populations.

A. Inflammatory cytokines cause human lung fibroblasts to overexpress *LRRC15*. Three public mRNA expression datasets were analyzed for expression changes upon the addition of cytokine cocktails including TGF-beta. The cell line and GEO identifier for each dataset is provided along the x-axis. The *GAPDH* gene is included as a negative control of a gene not expected to change in expression.

B. COVID-19 patients have large expansions of *LRRC15*-expressing fibroblasts in their lungs compared to uninfected patient controls. The percentage of lung fibroblasts where at least one mRNA count of *LRRC15* was detected is compared across a set of deceased COVID-19 patients and matched control lungs.

C. Lungs of COVID-19 patients experience a general shift toward more cells expressing *ACE2* and more cells expressing *LRRC15*. For the set of 7 main cell populations measured from human lungs, single-cell mRNA expression of *ACE2* and *LRRC15* is summarized between COVID-19 patients and controls.

When we explored the expression profile of lung tissue extracted from COVID-19 patients to matched control lungs, we further observed that there was greater expression (as measured by average mRNA counts and the percentages of cells that are *LRRC15*-positive) in *LRRC15* across multiple cell types. In the three primary lung cell populations we previously reported to express *LRRC15* (fibroblasts, endothelium, and some small epithelial populations), this new dataset that we have analyzed for our revisions shows all three show consistent increases in *LRRC15* levels in the lungs of COVID-19 patients (Figure R1C). Just as importantly, the expression of *ACE2* demonstrates a broadly similar pattern of increases. This both increases the co-localization of *ACE2* and *LRRC15* on lung cells and raises the plausibility of LRRC15 having significant functional consequences given its elevated expression.

The known low transcript abundance (and corresponding low detection rates) for *ACE2* and *LRRC15* make more detailed analyses challenging with the existing methods available for profiling expression. However, these analyses into some of the largest datasets that are currently public make *LRRC15* appear both more interesting and potentially relevant than our earlier figure considering only healthy lungs communicated. We thank the reviewers for suggesting what ended up being a productive line of analysis.

Major concerns

- Most data are from binding assays using soluble S protein. Fig 1E and Fig 2A show that LRRC15 interacts with S protein but very weakly. The authors suggest that this is due to low expression LRRC15 as binding is comparable to antibody staining (Fig 2A). The latter cannot be concluded as techniques are different (binding vs antibody binding, dimers etc). The authors could perform binding with actual SARS-CoV-2 particles to show that LRRC15 interacts with complete virus containing endogenous S protein. This would support the binding data obtained with S protein.

To clarify what we meant by the comparison to antibody staining, we are simply showing in Figure 2A that a majority of the cells we transfect or transduce to express LRRC15 do not end up expressing LRRC15 on their surface. We agree that technique differences between protein reagents and antibodies can result in varying staining profiles if they are compared quantitatively, but our intended comparison is purely qualitative. Our message is that there are only approximately 20% of *LRRC15*-transfected cells that have any detectable LRRC15 by antibody staining, and thus it is unsurprising that our binding experiments with spike protein are similarly limited so as to give as their maximum signal no more than approximately 20% of cells (since spike would not be expected to bind the population of cells that were refractory to transfection).

For the other part of the reviewer's question, data reporting that pseudotyped virus particles possessing the SARS-CoV-2 spike protein do bind to human cells expressing LRRC15 have now been reported by two separate preprints that were posted following the submission of our manuscript (Song *et al*, Loo *et al*). In the manuscript we now cite both preprints. We also describe in our response to comment 2 from Reviewer #2 the data that we have collected to

show attachment to LRRC15 by spike proteins in trimeric configurations, such as is seen on the natural virion surface.

> - The authors compare binding with other known receptors for SARS-CoV-2 and conclude that LRCC15 does not affect or induce binding to these receptors (line 161, fig 2). It is interesting to compare other receptors but it does not prove that LRRC15 binding is direct. Binding studies with recombinant LRRC15 and S protein (in ELISA for example) would support such a conclusion. This would also support the weak binding data observed (see above).

We agree with the principle that, whenever possible to perform, recombinant protein binding data provide a convincing demonstration of direct binding. Although we previously had been unable to produce a form of the truncated extracellular domain of LRRC15 that retains binding activity, we saw that in the months that followed the posting of our preprint, a separate group had replicated our results in a subsequent preprint that included tests with a recombinant LRRC15 protein generated by a commercial company (Song *et al*). We therefore sought to replicate the ELISA binding results described in that preprint in order to address this comment by the reviewer.

We purchased the same recombinant LRRC15-Fc protein (SinoBiological #15786-H02H) and followed their procedure of adsorbing spike protein to a MaxiSorp plate followed by testing a dilution series of recombinant LRRC15-Fc for direct binding in an ELISA-style experiment. When we did so, we were surprised to see that the recombinant LRRC15-Fc protein gave clear binding signals irrespective of the bait proteins immobilized onto the plate, including negative controls (Figure R2A). One interpretation of these data is that the commercially sourced LRR15-Fc protein contains a significant fraction of unfolded aggregated protein. To verify this was the fault of the recombinant LRRC15 reagent, we repeated the experiment, but this time captured biotinylated spike protein on a streptavidin-coated microtiter plate. The specificity of streptavidin's attachment to biotin greatly reduces the chances of non-specific protein adsorption compared to the MaxiSorp plates which may experience incomplete blocking. Again, the binding of the recombinant LRRC15-Fc reagent was indistinguishable between immobilized bait proteins, whether this was the SARS-CoV-2 spike protein or negative control proteins such as BSA. (Figure R2B). This confirmed that the recombinant LRRC15-Fc was associating non-specifically to the microtiter plate surface, which is a common issue with recombinant protein preparations that are not carefully prepared to ensure proper folding. A close inspection of the provenance of this reagent showed that it had been prepared as a lyophilized protein, which in our experience can increase the risk of the protein misfolding and binding non-specifically. We further noticed that the dissociation constant of the LRRC15-Fc reagent to blank wells closely matched the dissociation constant reported in that preprint, supporting the conclusion that recombinant LRRC15 in this formulation is unsuitable for binding assays.

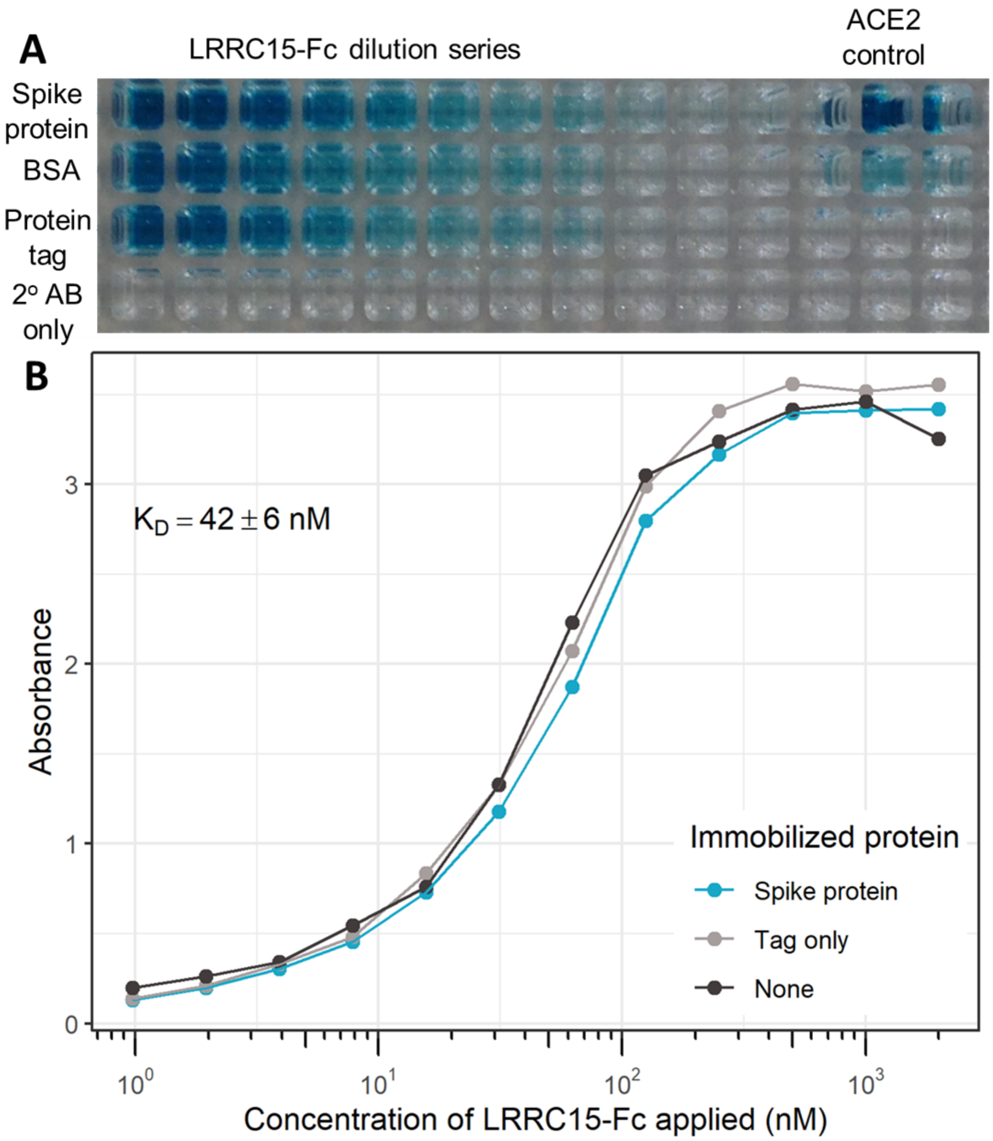

Figure R2. Assays utilizing commercial recombinant LRRC15 protein show evidence of non-specific binding, making them unusable for direct binding tests

A. Commercially-sourced recombinant LRRC15 Fc fusion protein shows evidence of non-specific binding to control protein baits immobilized on a MaxiSorp plate reveals non-specific binding. The photograph shows a 384-well plate where each row contains a different immobilized bait, and the columns are a dilution series of the recombinant LRRC15-Fc protein. A control well of ACE2 is applied on the right to verify the spike protein bait used is functional. Note that all immobilized bait proteins, including BSA and protein-tag-only negative controls, exhibit indistinguishable binding activity to LRRC15-Fc demonstrating its promiscuous binding activity in this assay.

B. Binding curves for a dilution series of recombinant LRRC15 on blocked streptavidin-coated plates continue to demonstrate non-specific binding. The same experiment as panel A was repeated on a streptavidin-coated plate, with binding curves quantified. The dissociation constant (K_D) calculated from the binding data is displayed in order to note that the calculated K_D

- even to the negative control proteins - has a similar value to that reported by the preprint study for specific binding to SARS-CoV-2 spike.

In the discussion section of our manuscript, we reflect on this finding since it suggests that the transmembrane and/or intracellular regions of LRRC15 are indispensable for the protein's proper presentation. Our findings that the truncated recombinant extracellular domain alone of LRRC15 does not support binding are supported by a recent study by an independent group which described an endogenous human protein (CD248) which binds human LRRC15 (Cao *et al.* 2021). Very similarly to the experiences described in our study on coronavirus spike binding to human LRRC15, they found that LRRC15 only displayed binding activity when presented as the full-length protein in the presence of a lipid bilayer membrane. We include a reference to Cao *et al* in our manuscript's discussion.

> - The authors state that the binding to LRRC15 is specific for SARS-CoV-2 but this is not correct and should be changed as suppl fig 5 shows that removal of glycans allows binding of LRRC15 with S protein from SARS-CoV-1. These data are very interesting and should be in main text, moreover, it suggest that the cell-lines and expression system might affect the binding of SARS-CoV-1 and SARS-CoV-2 as cell-lines have different glycosylation biases. Therefore would be interesting if the S protein from SARS-CoV-1 and -2 is overexpressed by more relevant cells such as lung epithelial cell-lines.

We were intrigued by the reviewer's suggestion to produce the spike proteins of SARS-CoV-1 and SARS-CoV-2 in a human lung epithelial cell line to try to resolve the role of spike glycosylation in the conservation of binding to LRRC15. We acquired the A549 human cell line, which are carcinomas derived from the same alveolar type II (AT2) lung epithelial cells that coronaviruses preferentially infect. This was a challenging proposition, as A549 cells are not typically employed for recombinant protein production and known as a "difficult to transfect" cell line (Sondergaard *et al* 2020). Nevertheless, we explored a range of different transfection and protein expression protocols to determine if A549 cells could express sufficient amounts of recombinant spike protein for our experiments.

After some optimisation, we eventually succeeded in transfecting and purifying constructs consisting of the full-length extracellular domains of both SARS-CoV-1 and SARS-CoV-2 (Figure R3A). However, we observed that the spike proteins produced by these cells did not appear at the expected molecular mass, and instead were detected as bands of smaller mass, most likely proteolytically processed products. Importantly, the sizes of these products were not consistent with the known proteolytic cleavage sites within the spike protein. If cleaved at the S2' site preceding the fusion peptide, the SARS-CoV-2 spike construct would be expected at a mass of 68 kDa. Cleavage at the S1/S2 would not be expected on any of these recombinant spike constructs since the polybasic region of the protein was mutated. By contrast, the most abundant protein form detected in the purification is at approximately 48 kDa, which is far too small to match the natural proteolytically processed form. Similarly the

SARS-CoV-1 spike would be expected to be above 68 kDa if it was cleaved at its S2' site, which does not match any of the bands observed on the protein gel.

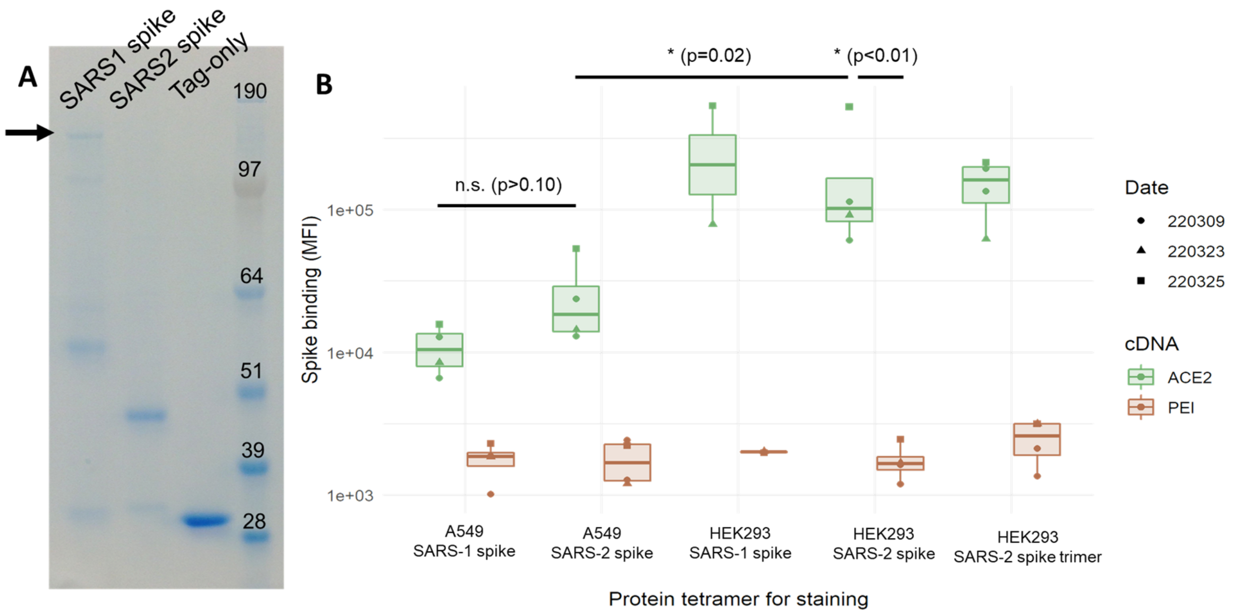

Figure R3. Recombinant spike protein can be produced from the A549 lung cell line, but with greatly diminished functional activity due to atypical proteolytic processing.
A. The human alveolar epithelium A549 cell line can be adapted to produce recombinant spike protein, but with atypical proteolytic processing. SARS spike proteins were expressed by A549 cells, purified, and resolved under reducing conditions by SDS-PAGE. The samples were stained with Coomassie blue dye to visualize the total protein content. The expected molecular mass for full-length SARS-CoV 1 or 2 spike is indicated by the black arrow. Masses are provided in units of kilodaltons (kDa).
B. Control tests of spike protein produced from A549 cells binding onto ACE2-expressing cells shows greatly diminished activity. Mean fluorescence intensity as measured by flow cytometry is shown for ACE2-expressing HEK293 cells (green) and mock-transfected HEK293 cells (red). The cell line used to produce each spike is listed along the x-axis above the spike protein name.

When, in spite of their unexpected masses, we tested the binding activity of these spike preparations to cells expressing LRRC15, the results were inconclusive. This was not unexpected given the nearly 100-fold diminished activity of the A549-produced spikes would make all but extremely strong binding signals difficult to differentiate from background staining. Although it would likely take an entire study in its own right to establish a robust method of isolating active spike protein from human type II alveolar cells, if such a method were invented we agree this would be an interesting question to re-visit, but unfortunately is beyond our current ability to produce spike proteins that are not unpredictably processed in ways that ablate their activity. In our revised manuscript, we devote more discussion to this role of glycan accessibility on the spike protein.

- The expression data using public data sets are overstated, even though there is a overlap in tissues between ACE2 and LRRC15, there is no overlap in lung cells. These data do not provide any relevant information. It would be more important to show whether ACE2 positive cells such as alveolar macrophages or epithelial cells (primary or cell-lines) express also LRRC15. The authors use standard cultured Calu-3 cells but these are very difficult to infect with SARS-CoV-2 due to low ACE2. Calu-3 cells need to be differentiated in monolayers to induce strong expression of ACE2 and expression of LRRC15 could be analysed in such a model. Even if there is no co-expression, LRRC15 could facilitate binding and transfer to other cells. But the co-expression needs to be more carefully investigated.

In our revised manuscript, we have amended this section of our results. First, as we describe above in response to the first comment by Reviewer #1, we have performed additional analyses into the overlap of LRRC15 and ACE2 in the lungs of COVID-19 patients. Second, we value the reviewer's insight that even in the absence of co-expression, another mechanism to explain how LRRC15 could modulate infection is through a *trans* mechanism where LRRC15-expressing cells influence viral entry into nearby ACE2-expressing cells. We test this mechanism in our revised manuscript to clarify the mode of LRRC15's effect. Those data can be found at the end of this document, corresponding to comment 5 by Reviewer #3 who asked a very similar question.

- Infection data using a recombinant SARS-CoV-2 is interesting and suggest that LRRC15 is not a receptor but could enhance infection. However, the enhanced infection of LRRC15-positive ACE2 cells is not very clear and lost when using higher MOI. Blocking reagents to LRRC15 (antibodies) would be useful to determine whether it is indeed the LRRC15 function that enhances infection. Here Calu-3 cells are not grown in a monolayer which affects infection. These experiments should be done on with differentiated Calu-3 cells in a monolayer as these are more susceptible to SARS-CoV-2 (and might already express LRRC15 but at the very least it is more relevant to SARS-CoV-2 infection).
Wild-type SARS-CoV-2 is highly infectious and infection experiments using SARS-CoV-2 isolates would support the observed data.

We thank the reviewer for their insightful suggestions on improving infection assays when done with CaLu-3 cells. Although we found our CaLu-3 cell protocol sufficient for generating high levels (>50%) of infection, the primary difficulty we encountered was fluctuations in ACE2 levels. This was the main impetus driving our experimental design, as we thought it was vital to ensure ACE2 levels were stable throughout our experiments so the effects of genetically manipulating LRRC15 were not confounded by ACE2 differences. In the experiments we have had added for our revisions where we test for the effects that LRRC15-expressing cells may have on modulating the infection of nearby ACE2-expressing

cells in *trans*, we have pursued an alternative highly sensitive infection model using the A549 cell line with a luciferase-based reporter that was recently established (Gerber *et al* 2022, Meng *et al* 2022).

As explained by Reviewer #2 in their comment below, we would also like to emphasize that the focus of our Short Report is to show that LRRC15 mediates an accessory interaction with the SARS-CoV-2 spike protein. Consequently, the manuscript is predominantly focused on characterizing the physical interaction with the viral spike protein. We included infection data with SARS-CoV-2 because we believed it was important to clarify that LRRC15 does not act as an entry receptor on its own but may modulate infection by other mechanisms. We have been pleased to see that subsequent to our posting of this discovery as a preprint, two further groups have followed up more on the potential mechanisms of this accessory interaction in modulating SARS-CoV-2 infectivity as the subject of their own preprints (Song *et al.*, Loo *et al.*) that build off from the foundational discovery and biochemical work we report in our manuscript. When considering the scope of our Short Report, we believe this achieves a reasonable balance of offering initial functional characterization data for the interaction in these two possible scenarios that are currently the topic of active research elsewhere.

minor comment

line 226-228. The sentence 'These findings suggest a novel function of the receptor-binding region of the SARS-CoV-2 spike protein which appears to be a relatively recent evolutionary innovation' is vague and not supported by the data.

In our revised manuscript, we have removed this speculative comment about evolution.

Reviewer #2: Coronavirus entry
Reviewer #2:

This submission demonstrates that SARS-CoV-2 spike protein ectodomain fragments bind a protein called LRRC15. This is a new discovery that adds one more spike-binding factor to several others that have been previously reported. The discovery of LRRC15 came from two well-executed genetic screens, and the subsequent documentation of LRRC15 as a spike-binding protein was convincingly demonstrated. The paper is strong for its thorough controls. However, the project and its impact is weakened with results that do not convincingly demonstrate a role for LRRC15 in virus infection.

1. Results in Fig 4E are used to claim a proviral role for LRRC15, but these are amongst the weakest data of the report. LRRC15 overexpression very modestly increases percentage of SARS2-positive Calu3 cells and only when virus is input at low input moi. At higher input moi, LRRC15 overexpression appears to have no effect. Also, increasing moi generated fewer infected cells overall. What explains this unusual

inverse correlation between input moi and cell infection? Also, Fig 4E expt and data could be expanded to determine whether LRRC15 accelerates infection, or could be expanded to other cell types beyond Calu3. In sum here, if the title of the paper remains as "LRRC15 mediates an "accessory" interaction"; and if the discussion keeps lines 326-327, that "LRRC15 modulates a host cells susceptibility to SARS-CoV-2" then further support of these claims, beyond Fig 4E, is recommended.

We appreciate the reviewer's suggestion for how the discussion should be rephrased to avoid the appearance of our study making stronger claims than we intended. The reviewer is very correct that the paper's title and majority emphasis remains simply that "LRRC15 mediates an accessory interaction with the spike protein", and our intention with this Short Report is to announce the discovery, provide detailed biochemical characterization of the novel interaction, and conclude by exploring the possibilities for potential functional roles LRRC15 may have in natural infection. In our revised manuscript, we have followed the reviewer's suggestion of replacing line 326 and more broadly have edited the final section of our manuscript addressing potential function with new data that explores both effects the spike protein's interaction with LRRC15 may have in *cis* (as we tested previously) as well as in *trans*. These new data can be found in our response to comment 5 by Reviewer #3, who suggested the specific functional experiment we subsequently performed.

In our revised results section, we have also proposed a model for how the concentration ("MOI") of virus present can be correlated to the magnitude of LRRC15's effect on infection. Briefly, we hypothesize that when virus is highly abundant, then it can efficiently enter through ACE2 irrespective of the presence of the comparatively weaker binding response the virus has to LRRC15. Similar concentration-dependencies were observed by another of the studies that was posted as a preprint, where they likewise saw that the highest virus MOIs they tested lead to dampened or absent effects on infection (Loo *et al*).

2. Binding data: The results include a great deal of binding data but do not include any data on the binding of complete virus particles to LRRC15. This seems like a major omission, particularly because spike trimers on virus particles have different conformation and glycosylation patterns than S1 or uncleaved S1-S2 ectodomains produced independent of virus infection (and given that glycosylation patterns appear to be central to LRRC15 binding, i.e., suppl fig 5).

For our revisions, we considered these confounding factors that could influence whether the spike protein on authentic SARS-CoV-2 particles would also bind onto LRRC15. As the reviewer delineates, the two main (although not exhaustive) reasons the spike proteins we used previously in our study could differ meaningfully from the spike protein found on virus particles would be differences in conformation stemming from the unique trimeric configuration of spike proteins on the viral surface, or differences in glycosylation based on our PNGase data. We tested each of these in turn.

First, we borrowed a previously-published design for making the spike protein assemble into a trimer by fusing its C-terminus to the T4 foldon trimerization domain (Li *et al* 2013, Hsieh *et al* 2020). This approach has been used in prior studies to achieve trimers that closely resemble the structure found on intact virions and displays a mix of both open and closed conformations (Wrapp *et al* 2020, Ke *et al* 2020, Walls *et al* 2020). After purifying trimeric SARS-CoV-2 spike protein based on this design, we performed cell-binding tests to gauge if attachment to LRRC15-expressing cells is preserved. The trimerized spike bound control cells that expressed ACE2 at similar levels to our previously-reported monomeric spike constructs, and also replicated the same clear binding to LRRC15-expressing cells we previously reported from our tests on monomeric spike (Figure R4).

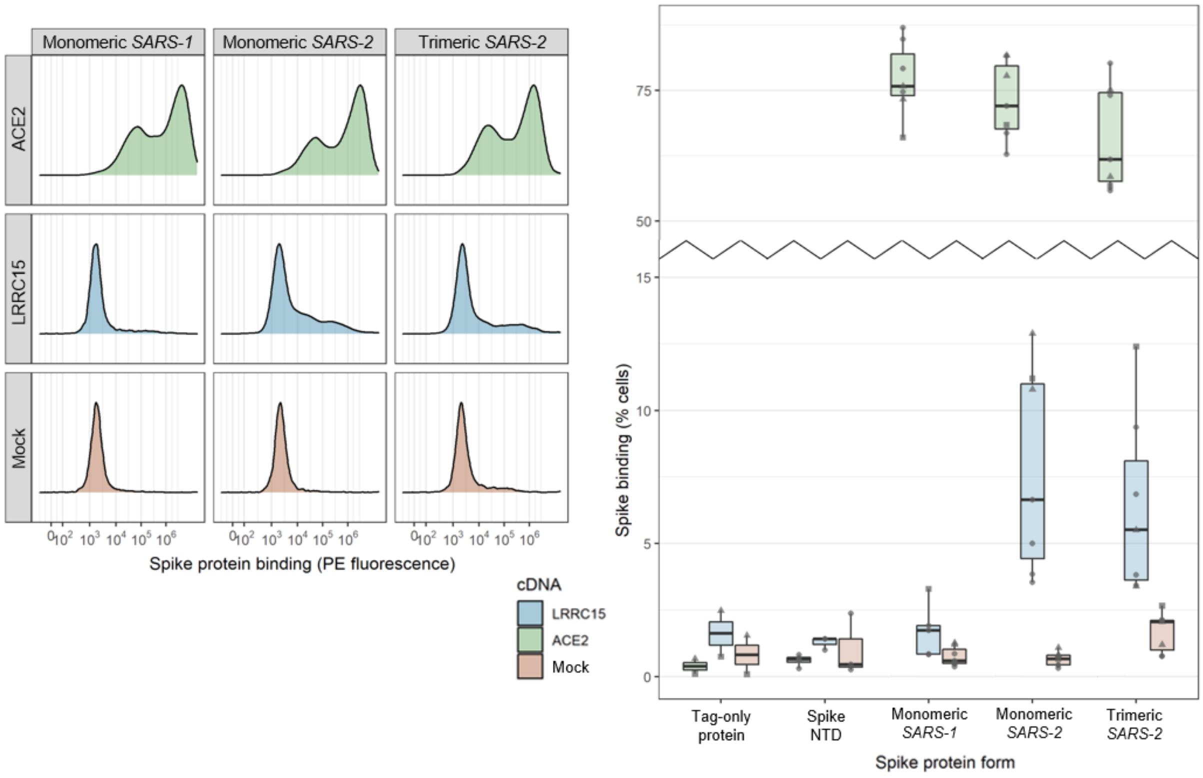

Figure R4 (*added the manuscript as the new Supplemental Figure 3)*. SARS-CoV-2 spike in a trimeric conformation binds LRRC15 at comparable levels to monomeric spike preparations.
A. Representative flow cytometry traces of HEK293 cells overexpressing the indicated cell surface protein (ACE2 in green, LRRC15 in blue, and mock-transfection control in red) stained by either monomeric or trimeric spike proteins.
B. Quantified binding of trimer spike protein, following from panel A. The y-axis is truncated at the indicated break point in order to display all conditions in a single plot.

For the possibility relating to glycosylation, we did expend considerable effort trying to produce recombinant spike protein from the A549 human alveolar cell line, but ultimately were unable to produce spike as we describe in Figure R3 above. We note that direct comparisons have already been reported between spike protein produced in HEK293 cells and spike proteins

on virions produced by human alveolar cells, which found high degrees of similarity (Yao *et al* 2020). Significantly, it was a *lack* of glycosylation that accounted for the gain of spike binding seen in our SARS-CoV-1 spike PNGase experiment. Therefore, LRRC15 recognition of spike does not appear contingent on narrowly specific glycans present on the spike but rather likely reflects a protein interface.

We were fortunate that in the time after this manuscript was submitted for review and posted as a preprint, two independent groups have subsequently studied the binding interaction between the SARS-CoV-2 spike protein and LRRC15 and performed the experiment requested by the reviewer. In the preprint posted by Song *et al*, they report measurements of pseudovirus particles attaching to LRRC15-expressing cells by two different methods (confocal microscopy and qPCR following wash steps to remove unbound viral particles). Consistent with our own data, they found clear evidence of viral attachment by both assays.

3. Coincidence of LRRC15 and ACE2 on target cells: Data show that only small cell proportions bind spikes, and this seems to hold for both LRRC15 binding and ACE2 binding. Are spike binding potentials of LRRC15 and ACE2 on the same small populations? If not, then what is the model for how LRRC15 is proviral?

The reviewer makes a reasonable observation that, based on our flow cytometry data measuring cell-surface staining against LRRC15 and ACE2, it would appear that for both only a small proportion of cells are stained. However, we believe that impression may be misleading due to sensitivity limitations for our staining protocol. To demonstrate this, consider the infection data we show in Figure 4, where the CaLu-3 target cells achieve infection rates averaging above 60% in the control, and reaching as high as 90% in individual experiments. From this it follows that at least 90% of the CaLu-3 cells must be ACE2-expressing and capable of binding spike, since only ACE2 (and not LRRC15) enables viral entry into the cell line. We suspect that the extremely low copy numbers of ACE2 receptors on the surfaces of the cells means that our flow cytometry measurements do not provide accurate absolute quantitation of the proportion of cells bearing ACE2. Rather, most CaLu-3 cells are ACE2-expressing. This discrepancy between the staining rate of methods like flow cytometry and microscopy to true ACE2 surface levels has also been reported by others using this cell line (Sherman *et al* 2021).

Therefore, our expectation is that the large majority of LRRC15-expressing cells in our experiment are also ACE2-expressing. In our model of LRRC15's effect on infection, we consequently propose that ACE2 and LRRC15 are both present on the cells which show enhanced infection. To further experimentally validate this model over an alternative model where LRRC15 and ACE2 do not have to be present on the same cells, in our revisions we co-cultured separate populations of purely LRRC15-expressing cells and purely ACE2-expressing cells to measure if the same proviral phenotype was observed. The data for this experiment are shown in Figure R10 under comment 5 from Reviewer #3. In brief, we did not find any evidence that LRRC15 on cells lacking ACE2 influences viral infection into nearby

ACE2-expressing cells. This provides further support for our model where ACE2 and LRRC15 co-expression are most likely to mediate LRRC15's effect on infection.

4. The discussion section makes several intriguing points about LRRC15 but (at least for this reviewer) does not put a clear view forward on LRRC15 activity in infection. One could envision abundant low-affinity binding agents capturing viruses at the same RBD sites that ACE2 uses for entry activation, and then presenting other presumably unbound viral spikes to ACE2 in some way, but this and alternative views are not provided. There are opportunities in the discussion to clarify how the LRRC15 might operate in infection, or, if the LRRC15 does not operate in infection but rather captures viruses "unproductively" then that could be communicated.

We are grateful for the reviewer's suggestion to consider "unproductive" virus attachment during infection as a possibility in our discussion. We have rewritten a portion of our discussion section to present this alternative possibility and critically discuss it. These suggestions by the reviewer are particularly timely because of the two preprints that were posted after this round of reviews for our manuscript, which both pose the hypothesis that LRRC15-expressing but ACE2-negative cells could unproductively capture virus particles. We elaborate on this possibility based on our own data testing this hypothesis (Figure R10), which as we just described ended up being negative and not finding evidence for this mechanism.

Reviewer #3: Receptor-ligand pathways

Reviewer #3:

In this manuscript, Dr. Wright and Lehner's groups have used their cutting-edge cell-based systemic screens to determine novel SARS-CoV-2 spike binding partners. They used ACE2 as a positive control for their screens, and identified LRRC15 as a new interaction partner specifically for the spike protein of SARS2, but not SARS1. A serial of delicate experiments were also performed to verify this interaction, particularly focusing on its difference with heparan sulfates or lectin receptors. The binding epitope was mapped to the c-terminus of S1. Functionally, similar to some of the other recently reported SARS2 interacting partners (lectins, LRP1 etc.), LRRC15 itself does not permit viral entry but may enhance ACE2-dependent SARS-2 infection as indicated by a reporter virus. Given the fact that LRRC15 is expressed in human lung vasculature cells and fibroblast, this interaction may have some implication in the physiological condition during SARS2 infection.

Overall, this finding is novel and the study was well-performed, particularly the systemic screens led by leaders in the field. The paper was also well-written. However, there are several technical concerns I see for potential improvement.

1. Other than ACE2, there are several S interaction partners have been recently identified (Several C-type lectins, LRP1, AXL, TLR4 etc). However, in the current

screens, only CLEC4M was used as positive control beside ACE2. Did both screens contain these reported spike binding partners? This is important as these information will validate the robustness of the screening systems described in this paper.

We agree that this is an important question to consider, both to gauge the sensitivity of the screening approaches we applied in our study as well as to have our systematic screens help clarify for the community which of the several claimed spike binding proteins appear to be broadly reproducible in different experimental formats. To answer this, we strove to measure the spike binding signal for not only the set of claimed receptors provided by the reviewer but also for any human cell-surface protein for which we could find at least one publication or preprint claiming it to have a direct binding interaction with the SARS-CoV-2 spike protein.

We compiled a list of 15 cell surface proteins for which we could find a claimed binding interaction. These proteins and their associated studies are : AXL from Wang *et al* 2021, LRP1 from Devignot *et al* 2022, BSG from Wang *et al* 2020, GRP78 from Carlos *et al* 2021, DPP4 from *Li et al* 2020, KREMEN1, KREMEN2, ASGR1, LILRB2, FUT8, LMAN2, SIGLEC9, and ERGIC3 from Gu *et al* 2021, TLR4 from Zhao *et al* 2021, and CNTN1 from Brockbank *et al* 2021. For each, we had full-length cDNA constructs available to express the receptor on our human cell lines. We individually tested each with an optimized low-throughput cell staining protocol and multiple measurements spanning 6-fold greater concentrations of spike protein than was used in our screens. These optimizations corresponded to control staining of nearly 10x greater intensity than the high-throughput screens, allowing us to ascertain whether there was any detectable binding signal.

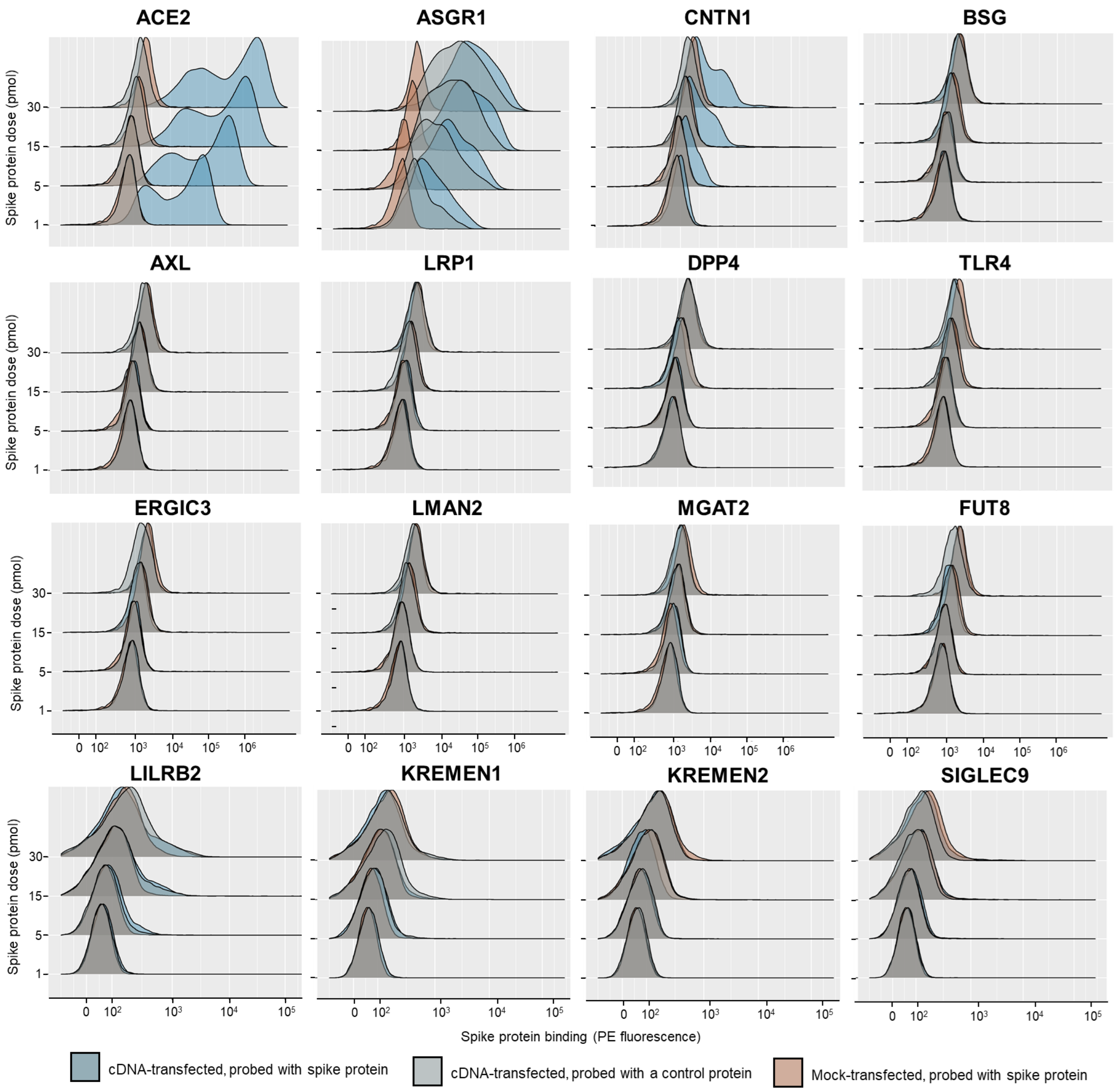

Figure R5. Independent testing of human receptors claimed by other studies to recognize the SARS-CoV-2 spike protein largely validates our screen's finding that most are not detectable. Each panel shows flow cytometry traces for HEK293 cells transfected with full-length cDNA for the indicated receptor. Four concentrations of spike protein (or a control protein of only epitope tags) were tested per condition. Of the fifteen tested spike-binding candidates, we observed detectable binding events for two (ASGR1 and CNTN1) using this assay.

For the vast majority of claimed receptors tested, 13 out of 15 gave no spike binding signal, consistent with the earlier systematic screen results (Figure R5). We did observe spike binding when HEK293 cells overexpressed ASGR1, although notably we would classify this signal as a false positive according to our screening criteria because nearly-identical binding

signal was observed for ASGR1-expressing cells binding to a negative control protein (consisting only of protein epitope tags and a fragment of rat Cd4, which contains a single predicted N-linked glycosylation sequon). This is not unexpected because ASGR1 is a C-type lectin and known to bind a broad range of glycan moieties in a calcium-dependent manner. To confirm that this binding is mediated through ASGR1's known lectin activity and to offer an explanation why it was not detected during screening, we experimented with depleting the calcium ions required for ASGR1 binding to glycans. Even without the addition of EDTA, simply using PBS that is not supplemented with millimolar concentrations of Ca^{++} was sufficient to prevent all binding (Figure R6). The binding results from overexpressing another candidate receptor, LILRB2, led to a very minor binding shift, but similarly it was indistinguishable from binding to the negative control protein.

This both verifies that the binding is likely not a direct protein-protein interaction and could explain how the binding was missed given it is highly dependent on calcium levels even in the absence of any chelator being added. Other known c-type lectins, including CLEC4M, also have binding activity that is dependent on the presence of metal cation cofactors, which may explain why CLEC4M was only detected in our arrayed screen but not in the CRISPR-activation screen.

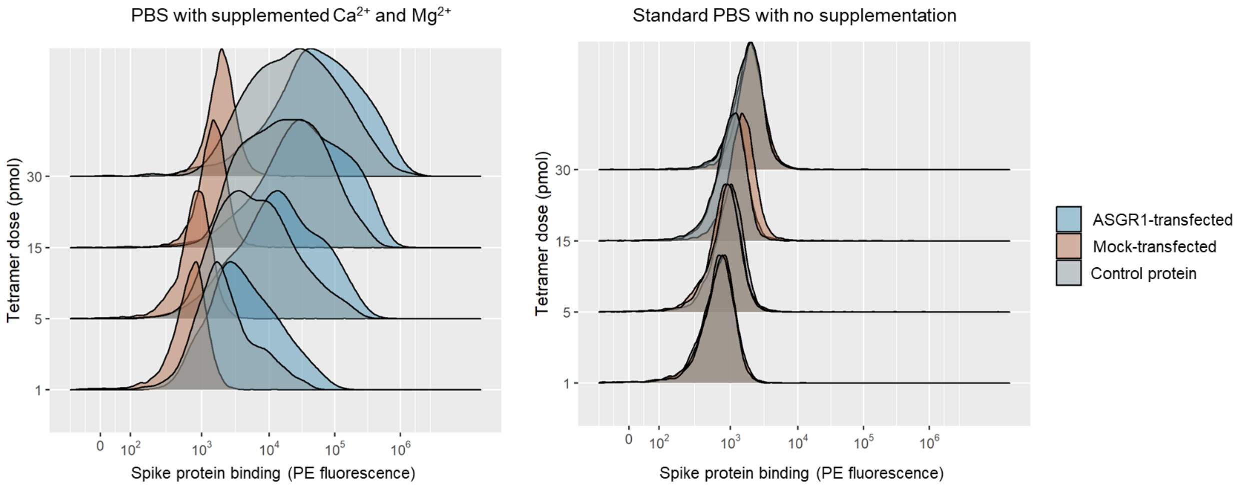

Figure R6. Spike protein binding to the ASGR1 receptor is highly sensitive to the media formulation used during testing.
Comparison of flow cytometry binding results achieved when conducting the experiment with two different formulations of PBS - one expressly supplemented with divalent cations (left) and the other with no supplementation specified (right). ASGR1 (Asialoglycoprotein receptor 1) is a known C-type lectin and we show that cells overexpressing ASGR1 bind to a glycosylated control protein as well as spike in a divalent-cation dependent manner.

The sole exception out of the set of these claimed receptors we investigated was CNTN1. Consistent with what was described in a recent unpublished preprint (Brockbank *et al*),

we could observe binding of the SARS-CoV-2 spike protein to human cells overexpressing CNTN1. At the quantities of spike protein used our initial screening (5 pmol), the binding shift was subtle, which likely accounts for why it was missed not only by our screens but also by both of the other recent unpublished preprints that conducted genome-wide screens which replicated our findings on LRRC15 but failed to identify CNTN1 (Loo *et al* and Song *et al*). Although the study by Brockbank *et al* did not focus on CNTN1 in their results, in one follow-up experiment they did determine that spike binding to CNTN1 appeared very weak, having a binding EC_{50} value above 150 μg/mL (with the precise value too large for them to quantite). Thus whether the binding event with CNTN1 is strong enough to be physiologically-significant remains unclear.

We should emphasize that our tests here do not, and can not, “prove” a negative result. It is possible that some of these proteins may enable attachment to the SARS-CoV-2 spike protein, such as if the proteins must be presented in a specific way or environmental context. Exhaustively testing even in a single claimed interaction across a wide range of experimental contexts in an attempt to demonstrate a negative interaction takes a great deal of effort. For example, our laboratories have previously completed a detailed study evaluating one such claim in the literature that basigin (BSG/CD147) could bind the SARS-CoV-2 spike protein. This study required months of work and was the subject of an entire publication to more confidently say our lack of binding spike signal appears to be a true negative and not merely a false negative from the techniques we employed (Shilts *et al* 2021). Therefore although a thorough accounting of these 15 other claimed spike binding receptors would require at minimum 15 entire separate publications, for the purposes of this study we believe our results underline the high sensitivity of our screening approach. We further believe this emphasizes the value of making our systematic screening data available to the community, to help resolve these important controversies around whether alternative SARS-CoV-2 spike-binding receptors exist.

2. The LRRC15 binding data look quite weak in general, even much weaker than CLEC4M (Fig 1C), which may because of 1) it is a weak interaction or 2) the insufficient expression of LRRC15 on the cell-surface (Fig 2A for instance). The authors may need to establish a LRRC15 high-expression cell line and then test the binding. Alternatively, are there any LRRC15 positive cancer cell lines can be used for this purpose? Moreover, the authors used a cell-based titration system to monitor the KD and found it was around 260nM. However, this system is not accurate. The authors need to at least test the direct protein-protein interaction of Spike/LRRC15 ECD by ELISA or Octet to calculate the more reliable KD.

This was a very reasonable suggestion by the reviewer to get stronger binding signals by utilizing an alternative cell line that endogenously expresses LRRC15 at high levels. To attempt this, we began by analyzing a database of mRNA expression values that spans 1,293 human cell lines (Cell Model Passports, Van der Meer *et al* 2019). When we ranked these by *LRRC15* levels, we identified the glioblastoma cell line U118-MG as high levels of *LRRC15* mRNA detected, as well as being unambiguously negative for *ACE2* and *CLEC4M* expression (Figure R7A).

Although not a widely distributed cell line, we managed to acquire U118-MG cells and could culture them as previously described (Figure R7B). However, when we stained these cells with anti-LRRC15 antibody, we could observe no cell-surface LRRC15 (Figure R7C). Possible explanations for this inconsistency include differences in the U118-MG stock we received compared to the exact line measured in the Cell Model Passports, that this cancer cell line does not properly synthesize or export LRRC15 protein to its surface, or a poor correlation between mRNA transcript and protein levels. This is in contrast to the known localization on more physiologically relevant cell types like normal human lung fibroblasts, which are described as having LRRC15 expressed on their surface similarly to our HEK293 cell model (Purcell *et al* 2018).

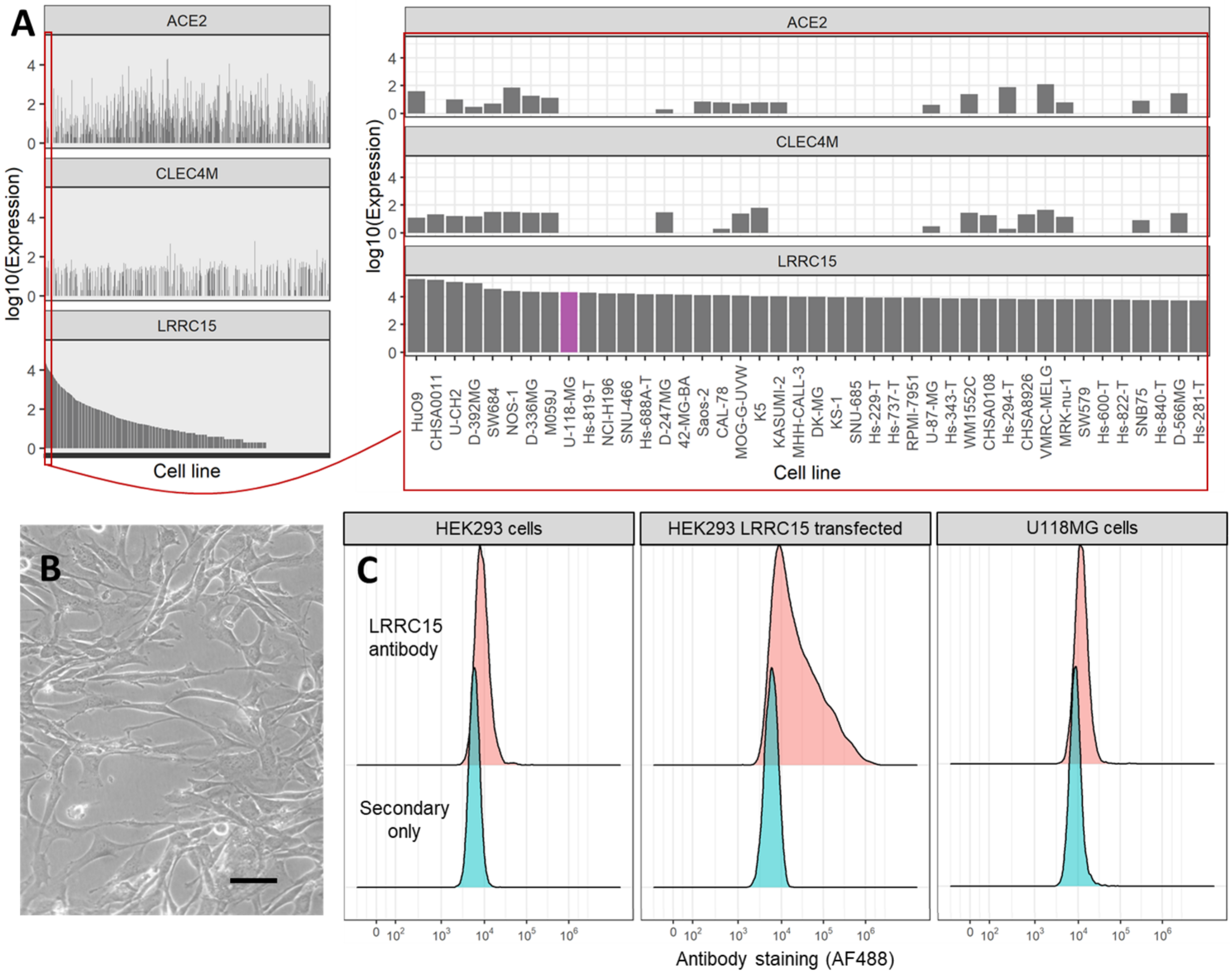

Figure R7. Attempts to identify a cancer cell line with high LRRC15 levels failed to detect LRRC15 expression by flow cytometry.

A. The Cell Model Passports database suggests cancer cell lines that may express LRRC15 at high enough levels to be useful as an experimental model. An overview of the more than one thousand cell lines in the database is provided (left) alongside an inset that zooms in on the cell

lines with the highest reported LRRC15 levels according to RNA-seq. The U-118 MG cell line, which was selected for further experimental testing, is highlighted in purple.
B. U-118 MG glioblastoma cells were acquired and grew with their expected morphology. A brightfield microscopy image is shown for adherent U-118 MG cells. Scale bar represents 100 µm.
C. There is no detectable LRRC15 on the surface of glioblastoma U-118 MG cells. Flow cytometry traces for staining with a monoclonal anti-LRRC15 antibody (top) or secondary-only control antibody (blue) are shown.

To address the last part of the reviewer's question dealing with recombinant protein-based binding assays, in our revisions we have performed binding tests using recombinant LRRC15. In doing so we attempted to exactly replicate the result of a preprint that was posted subsequent to our manuscript's submission (Song *et al*), that shows ELISA for measuring the K_D of LRRC15 binding to spike protein exactly as was requested above. However, as explained in detail in our first comment to the first reviewer (Figure R2), we found that the LRRC15 recombinant protein reagent that is commercially available (and used in this preprint) binds non-specifically to plastic substrates, making it unusable for specific binding experiments. Despite all our best attempts, it does not appear that artificially truncating the extracellular domain of LRRC15 to make a recombinant protein will retain binding activity. We elaborate on this in our Discussion section, which references another recent study by Cao *et al* (2021) which found a human binding partner for LRRC15 and similarly could only observe binding in the context of a cell membrane, not for soluble recombinant proteins.

While this means the specific techniques recommended by the reviewer are not possible to perform, we would emphasize the evidence supporting the accuracy of our cell-based K_D measurement. We scrupulously followed the guidelines published in an influential review by Hunter and Cochran (2016), which discusses the mathematics behind calculating binding equilibrium dissociation constants using cell-based binding assays, and how to ensure that the experimental setup satisfies all the assumptions of those equations in order to yield an accurate K_D value. One of the pre-prints validating our discovery of LRRC15 as a spike binding partner (Loo *et al*) independently performed a cell-based binding assay to measure the strength of binding and calculated a similar value of approximately 100 nM. As an additional layer of confidence, this is also why we include supplemental figure 3, which shows using our cell-based assay yields an equilibrium dissociation constant for ACE2 - spike binding in full agreement with published values calculated by methods such as BLI or SPR.

3. The HSPG and EDTA data is quite interesting, which indicate that LRRC15 binding is likely different from HSPG/Lectin binding of SARS2 spike. Does this interaction glycan dependent, or involve any glycosylations sites in the spike? The authors claimed that this interaction differs from previously described spike-binding receptors, however, which is quite over-stated. The authors only mapped this interaction to the CTD of Spike, how about RBD (major binding interface for ACE2 binding) and the rest of the CTD (which are mostly named as CTD)? The CTD region is quite big, and the authors need more

detail mapping to locate the exact binding region of LRRC15. Moreover, ACE2 competition data may have other interpretation beyond sharing the same epitope---such as steric hindrance.

We agree that it would be valuable to further localize the exact binding region on the spike protein that mediates the attachment to LRRC15 in order to clarify these alternative hypotheses for our data. We designed an expression construct consisting of only the receptor binding domain (RBD) for ACE2 (Figure R8A). This is 40% smaller than the spike S1 C-terminal domain construct we previously used (R319 - F541 versus R319 - Y674). We observed that the RBD region of the spike protein alone was sufficient to account for all of the binding signals seen with the larger C-terminal S1 domain truncation (Figure R8B-C). This supports our earlier data suggesting ACE2 and LRRC15 compete for binding.

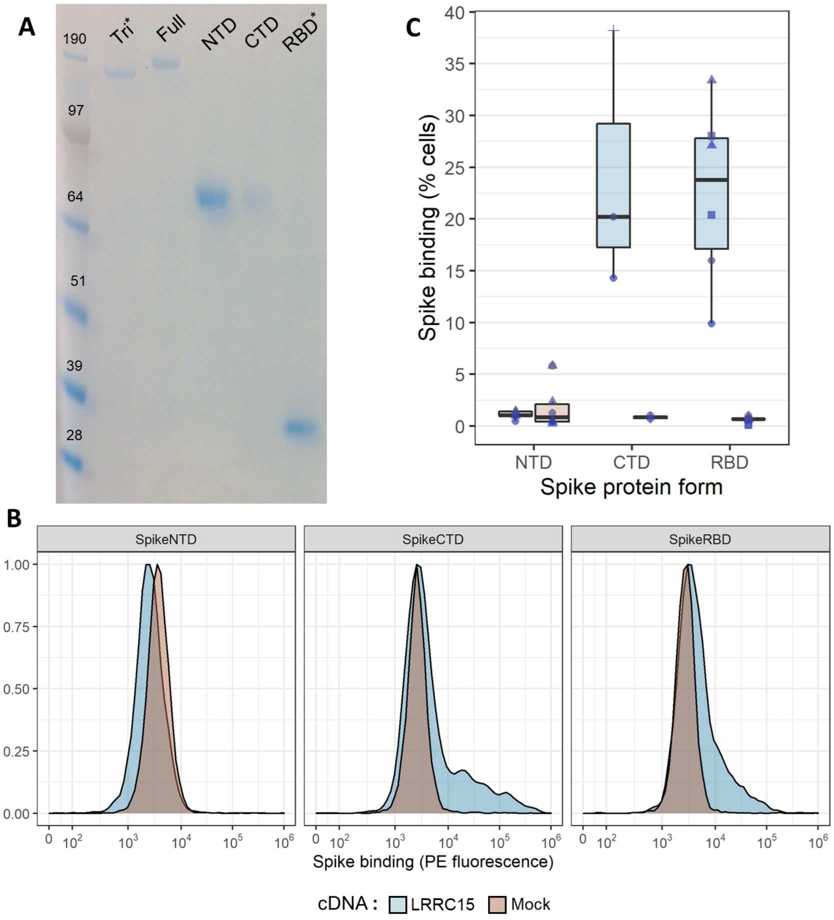

Figure R8 (*added to the revised manuscript in main figure 3*). The LRRC15-binding domain of the SARS-CoV-2 spike protein localizes specifically to the spike's receptor binding domain (RBD).

A. Expression of SARS-CoV-2 spike protein truncations to test for binding to the RBD. The entire ectodomain of SARS-CoV-2 spike proteins expressed as both a trimer (Tri) and monomer (Full) and the indicated truncations were expressed in HEK293 cells, purified, resolved by

SDS-PAGE under reducing conditions, and stained with Coomassie blue dye. The two constructs with an asterisk* have a shorter protein-epitope tag fused to them than the others (~5 kDa versus 28 kDa). Molecular masses are indicated in units of kilodaltons (kDa).
B. Representative flow cytometry traces show the RBD of SARS-CoV-2 accounts for all the binding activity of the spike C-terminal domain (CTD). LRRC15-transfected HEK293 cells (blue) were compared to mock-transfected cells (red). The spike N-terminal domain (NTD) of the S1 domain was included as a control to replicate our previously-observed lack of binding.
C. Quantified binding activity of spike S1 domain truncations, following from panel B.

We have added these data to the revised manuscript as a new panel in Figure 3 along with a description in the results of how these data agree with our earlier finding that LRRC15 and ACE2 compete for binding. The localization of LRRC15 binding down to the spike RBD that we had added in these revisions is supported by data presented in both of the two preprints that subsequently reported this interaction, as they likewise show binding localized to the RBD (Loo *et al*, Song *et al*).

> 4. As LRRC15 does not support SARS-2 infection, however, the authors found that LRRC-15 overexpression in Calu-3 cells slightly increase SARS-reporter virus infection. This data is important but need some controls--for example, did LRRC15 affect ACE2 expression? How strong the expression of LRRC15 can lead to more SARS-2 infection in this system? Moreover, given that LRRC15 is expressed in lung vascular/lymphatic endothelium or fibroblast cells, what is the percentage of LRRC15+ cells that are ACE2 positive (and vice versa)? It seems that the chance of co-expression of ACE2 to LRRC15 is quite low, which may affect the interpretation of the importance of this study, particularly in the physiological condition.

In recognition of the importance of confirming that LRRC15 does not affect ACE2 expression levels as the reviewer correctly points out, we include several controls in our manuscript. Most prominently, we performed sensitive western blots on the CaLu-3 cells we used as our infection model in Figure 4, which can be found in Figure 2F of our manuscript. In CaLu-3 cells where LRRC15 was genetically upregulated, ACE2 levels were comparable to unmodified control cells. For our revisions we also include data measuring cell-surface ACE2 levels by flow cytometry. Although the native levels of ACE2 on our CaLu-3 cells are very low (as evinced by the long exposure time being required on the western blot), we could nevertheless see that even very large upregulation of LRRC15 (>20-fold mean fluorescence intensity increase) does not produce any appreciable change in ACE2 levels (Figure R9).

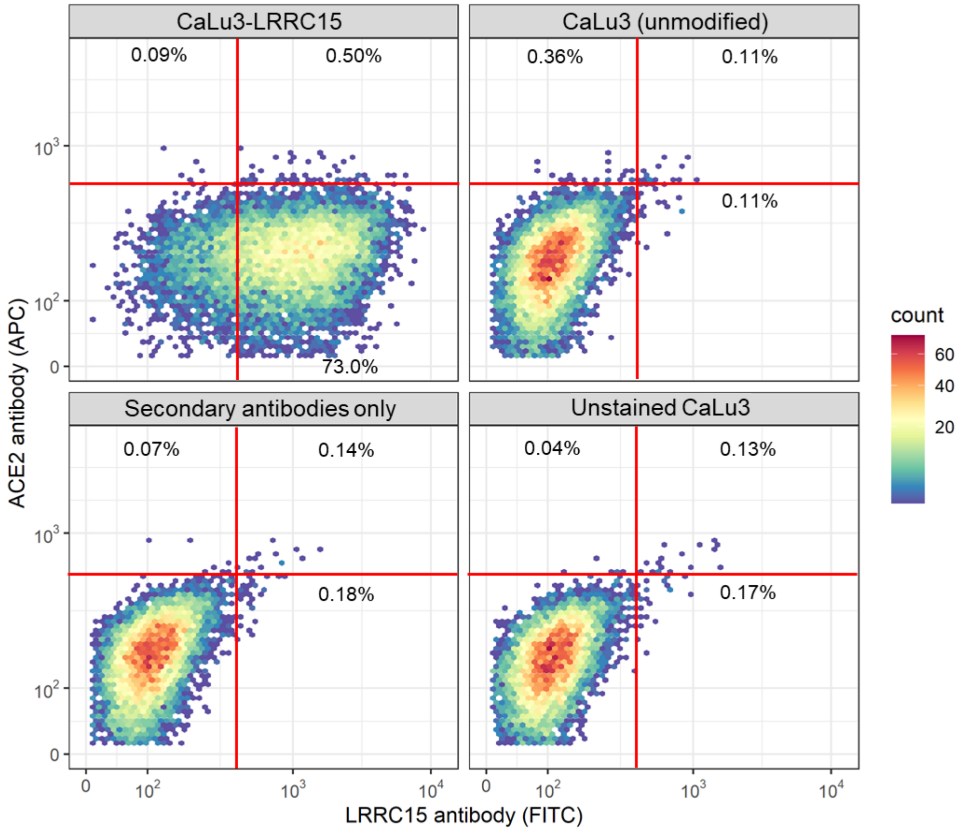

Figure R9. CaLu-3 cells transduced to express LRRC15 do not exhibit noticeable changes in cell-surface ACE2 levels.
Flow cytometry dot plots measuring both ACE2 and LRRC15 protein levels on the surface of CaLu-3 cells transduced with lentivirus encoding full-length *LRRC15* and controls. Bisecting gates are overlaid along with their corresponding percentages of cells.

Regarding the frequency at which ACE2 and LRRC15 co-expression occurs in the human lung, in the revised manuscript we have adjusted our wording in this final section addressing possible infection phenotypes. We address this question through our new analysis of public datasets of LRRC15 and ACE2 expression in the lungs of COVID-19 patients, as we described above for the first comment by Reviewer #1. The question of co-expression also relates to the reviewer's question about *trans* infection (since a *trans* mechanism would obviate the need for co-expression), which we discuss in the next section.

5. C-type lectins have been suggested to support SARS2 transinfection, how about LRRC15?

This was an excellent question, and also directly relates to several other helpful suggestions we received from the other two reviewers. In our original manuscript we had only considered *cis* effects of LRRC15 on cells co-expressing ACE2. In our revised manuscript, we now offer a more complete perspective that considers both possible mechanisms by which

LRRC15 could influence infection: either in *cis* (as we previously tested) or in *trans* (as our new data tests).

In the context of this question, an additional development that motivates these experiments is that in the months after we submitted our manuscript, two separate research groups independently replicated our core findings about LRRC15 and also included additional experiments testing *trans* infection phenotypes. Both studies reported that LRRC15-expressing cells may act as an unproductive “sink” that binds onto virions but does not permit entry, thereby inhibiting the infection of nearby ACE2-expressing target cells. Crucially, both of these preprint studies formulated this hypothesis on the basis of pseudovirus infection experiments. So although a fascinating hypothesis, neither have conclusively verified this *trans* occurs with infection by authentic SARS-CoV-2. Therefore, in our revised manuscript we took the prerogative of experimentally testing whether LRRC15-expressing cells influence the infectivity of authentic (i.e. non-pseudotyped) SARS-CoV-2 by a *trans* mechanism.

We co-cultured HEK293T cells containing a genomically-integrated construct for doxycycline-inducible expression of LRRC15 with ACE2-expressing human lung epithelial cells (Figure R10A). Following advice from Reviewer #1 about some potential limitations of CaLu-3 cells as an infection model, we instead utilized A549 cells, which are derived from human type II alveolar epithelial cells (Rihn *et al* 2021). We also selected this cell model because it has low expression levels of ACE2, which may make it easier to observe a *trans* effect. For achieving a highly sensitive readout of infection, we used a recently-developed reporter system where SARS-CoV-2 viral proteases amplify a luciferase signal in cells that are infected (Gerber *et al* 2022, Meng *et al* 2022). We verified that we could readily induce LRRC15 expression with minimal levels of expression leakage in the uninduced state (Figure R10B). For every co-culture experiment, 10,000 A549 target cells were mixed with 30,000 HEK293T cells. The fraction of HEK293T for each expression condition was varied across 4 levels, and the total was brought up to 30,000 by adding unmodified HEK293T cells. We verified the experimental setup in a control test showing that co-culturing A549 target cells with constitutively ACE2-expressing HEK293T cells resulted in suppressed infection rates of the A549 cells (Figure R10C).

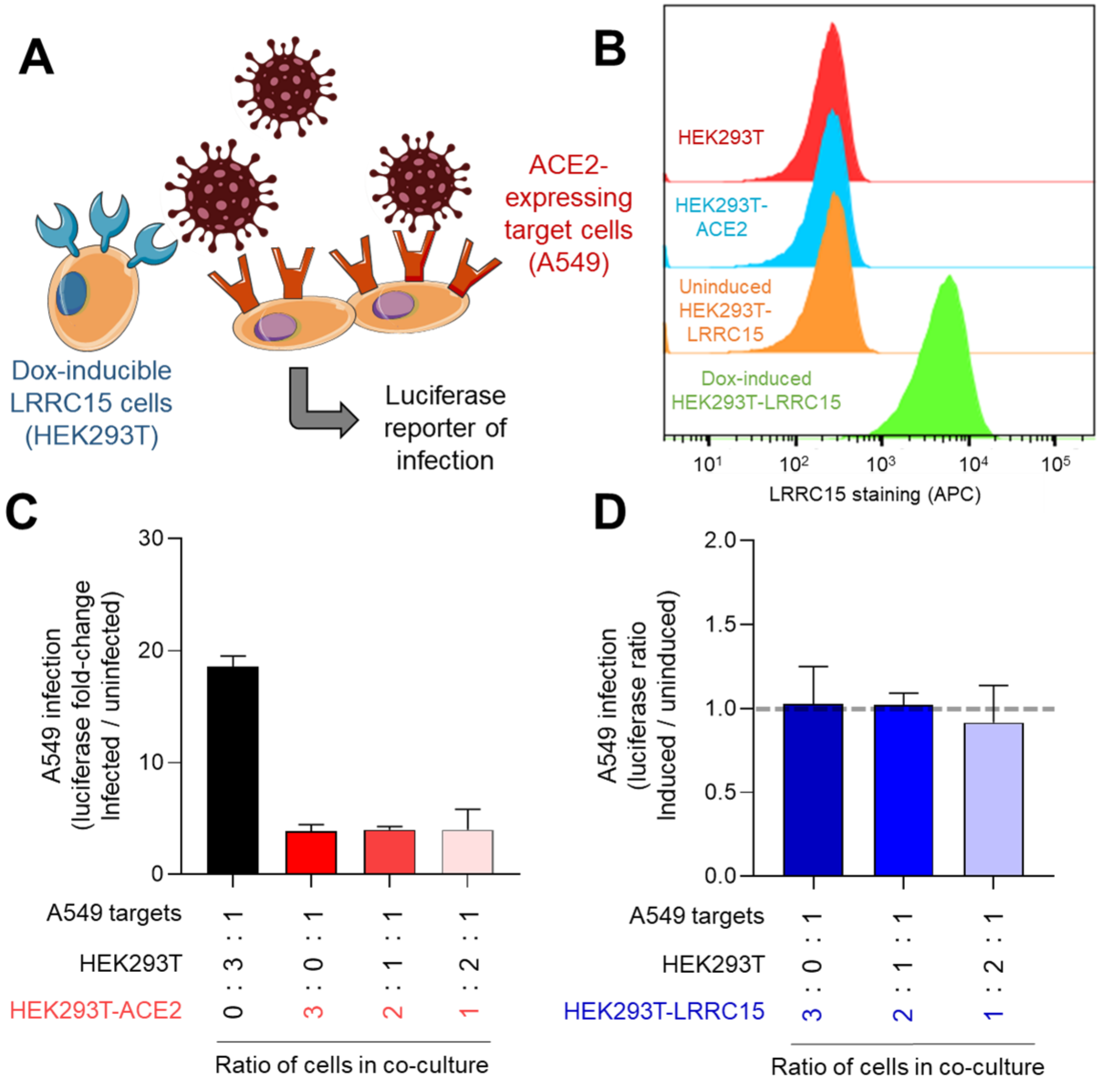

Figure R10 (*added to the manuscript as supplemental figure 9*). Co-culturing ACE2-expressing A549 respiratory epithelial cells with LRRC15-expressing HEK293T cells does not appreciably change rates of infection.

A. Schematic of the experiments. The A549 target cells are respiratory epithelium that express ACE2 and a luciferase-based biosensor of SARS-CoV-2 infection. HEK293T cells express doxycycline-inducible LRRC15.

B. Surface LRRC15 expression can be readily induced by doxycycline with minimal background staining in the uninduced state. Flow cytometry traces show HEK293T cells in different expression conditions (blue for constitutive ACE2 expression, orange for uninduced LRRC15, green for induced LRRC15 expression, and red for unmodified HEK293T cells).

C. Control tests of co-culturing A549 target cells with ACE2-expressing HEK293T cells show a large suppression of target cell infection. A549 respiratory epithelial cells expressing ACE2 and a luciferase-based biosensor of SARS-CoV-2 infection (A549 targets) were mixed with different ratios of control HEK293T cells or HEK293T cells expressing ACE2 (HEK293T-ACE2), then infected with SARS-CoV-2. Infection of A549 targets is quantitated as the fold-change in

luciferase activity in infected versus uninfected cells. Mean values ± SD are shown for an experiment performed in triplicate, representative of 2 independent experiments.
D. Induction of LRRC15 on HEK293T cells in co-cultures with A549 cells results in minimal differences in infection of the susceptible A549 target cells. A549 respiratory epithelial cells expressing ACE2 and a luciferase-based biosensor of SARS-CoV-2 infection (A549 targets) were mixed with different ratios of control HEK293T cells or HEK293T cells expressing doxycycline-inducible LRRC15 (HEK293T-LRRC15) in the presence or absence of doxycycline, then infected with SARS-CoV-2. Infection of A549 targets is quantified as the ratio of luciferase activity in conditions with (LRRC15 high) or without (LRRC15 low) doxycycline induction, with a ratio of 1 implying no effect of LRRC15 expression. Bars show the pooled standard error around the mean value from experiments done in triplicate, representative of two independent experiments.

We detected no difference in infectivity between A549 cells cultured in the presence of cells induced to express LRRC15 and matched cells that were uninduced (Figure R10D). In our revised manuscript, we discuss these results as implying that if the *trans* inhibition in infection reported by the other two studies does take place, then our own data suggests the conditions under which this phenomenon occurs may be limited to relatively specific contexts. We believe our use of an authentic isolate of SARS-CoV-2 for infection measurements makes our findings particularly informative when considering whether the results reported in those preprint studies that were gathered largely from artificial pseudotype systems are likely to hold in more realistic settings. Although the data we observed when performing the experiments requested by the reviewer ended up being negative, we nevertheless believe that they are very valuable contributions to the research community's understanding of in which circumstances LRRC15 can modulate infection and in which it may not. While the focus of the Short Report we submitted has always been to report the discovery and characterization of the binding interaction between LRRC15 and the SARS-CoV-2 spike protein, we believe that these additional infection experiments we performed to assess both *cis* and *trans* mechanisms on viral infectivity make an important contribution toward the growing interest (and debate) over LRRC15's role in coronavirus biology that is now the focus of several other ongoing studies.

References cited

Brockbank SM, Soden J, Faba-Rodriguez R, Ribeiro LR, Geh C, Thomas H, et al. SARS-CoV-2 comprehensive receptor profiling: mechanistic insight to drive new therapeutic strategies. bioRxiv; 2021. p. 2021.03.11.434937. doi:10.1101/2021.03.11.434937

Cao S, Peterson SM, Müller S, Reichelt M, McRoberts Amador C, Martinez-Martin N. A membrane protein display platform for receptor interactome discovery. Proc Natl Acad Sci U S A. 2021;118: e2025451118. doi:10.1073/pnas.2025451118

Carlos AJ, Ha DP, Yeh D-W, Van Krieken R, Tseng C-C, Zhang P, et al. The chaperone GRP78 is a host auxiliary factor for SARS-CoV-2 and GRP78 depleting antibody blocks viral entry and infection. J Biol Chem. 2021;296: 100759. doi:10.1016/j.jbc.2021.100759

Devignot S, Sha TW, Burkard T, Schmerer P, Hagelkruys A, Mirazimi A, et al. Low Density Lipoprotein Receptor-Related Protein 1 (LRP1) is a host factor for RNA viruses including SARS-CoV-2. bioRxiv; 2022. p. 2022.02.17.480904. doi:10.1101/2022.02.17.480904

Gerber PP, Duncan LM, Greenwood EJ, Marelli S, Naamati A, Teixeira-Silva A, et al. A protease-activatable luminescent biosensor and reporter cell line for authentic SARS-CoV-2 infection. PLoS Pathog. 2022;18: e1010265. doi:10.1371/journal.ppat.1010265

Gu Y, Cao J, Zhang X, Gao H, Wang Y, Wang J, et al. Receptome profiling identifies KREMEN1 and ASGR1 as alternative functional receptors of SARS-CoV-2. Cell Res. 2022;32: 24–37. doi:10.1038/s41422-021-00595-6

Hsieh C-L, Goldsmith JA, Schaub JM, DiVenere AM, Kuo H-C, Javanmardi K, et al. Structure-based design of prefusion-stabilized SARS-CoV-2 spikes. Science. 2020;369: 1501–1505. doi:10.1126/science.abd0826

Hunter SA, Cochran JR. Cell-Binding Assays for Determining the Affinity of Protein-Protein Interactions: Technologies and Considerations. Methods Enzymol. 2016;580: 21–44. doi:10.1016/bs.mie.2016.05.002

Ke Z, Oton J, Qu K, Cortese M, Zila V, McKeane L, et al. Structures and distributions of SARS-CoV-2 spike proteins on intact virions. Nature. 2020;588: 498–502. doi:10.1038/s41586-020-2665-2

Li J, Ulitzky L, Silberstein E, Taylor DR, Viscidi R. Immunogenicity and protection efficacy of monomeric and trimeric recombinant SARS coronavirus spike protein subunit vaccine candidates. Viral Immunol. 2013;26: 126–132. doi:10.1089/vim.2012.0076

Li Y, Zhang Z, Yang L, Lian X, Xie Y, Li S, et al. The MERS-CoV Receptor DPP4 as a Candidate Binding Target of the SARS-CoV-2 Spike. iScience. 2020;23: 101160. doi:10.1016/j.isci.2020.101160

Loo L, Waller MA, Cole AJ, Stella AO, Moreno CL, Denes CE, et al. LRRC15 suppresses SARS-CoV-2 infection and controls collagen production. bioRxiv; 2021. p. 2021.11.09.467981. doi:10.1101/2021.11.09.467981

Meng B, Abdullahi A, Ferreira IATM, Goonawardane N, Saito A, Kimura I, et al. Altered TMPRSS2 usage by SARS-CoV-2 Omicron impacts infectivity and fusogenicity. Nature. 2022;603: 706–714. doi:10.1038/s41586-022-04474-x

Purcell JW, Tanlimco SG, Hickson J, Fox M, Sho M, Durkin L, et al. LRRC15 Is a Novel Mesenchymal Protein and Stromal Target for Antibody-Drug Conjugates. Cancer Res. 2018;78: 4059–4072. doi:10.1158/0008-5472.CAN-18-0327

Rihn SJ, Merits A, Bakshi S, Turnbull ML, Wickenhagen A, Alexander AJT, et al. A plasmid DNA-launched SARS-CoV-2 reverse genetics system and coronavirus toolkit for COVID-19 research. PLoS Biol. 2021;19: e3001091. doi:10.1371/journal.pbio.3001091

Sherman EJ, Emmer BT. ACE2 protein expression within isogenic cell lines is heterogeneous and associated with distinct transcriptomes. Sci Rep. 2021;11: 15900. doi:10.1038/s41598-021-95308-9

Shilts J, Crozier TWM, Greenwood EJD, Lehner PJ, Wright GJ. No evidence for basigin/CD147 as a direct SARS-CoV-2 spike binding receptor. Sci Rep. 2021;11: 413. doi:10.1038/s41598-020-80464-1

Søndergaard JN, Geng K, Sommerauer C, Atanasoai I, Yin X, Kutter C. Successful delivery of large-size CRISPR/Cas9 vectors in hard-to-transfect human cells using small plasmids. Commun Biol. 2020;3: 319. doi:10.1038/s42003-020-1045-7

Song J, Chow RD, Pena-Hernandez M, Zhang L, Loeb SA, So E-Y, et al. LRRC15 is an inhibitory receptor blocking SARS-CoV-2 spike-mediated entry in trans. bioRxiv; 2021. p. 2021.11.23.469714. doi:10.1101/2021.11.23.469714

Van der Meer D, Barthorpe S, Yang W, Lightfoot H, Hall C, Gilbert J, et al. Cell Model Passports-a hub for clinical, genetic and functional datasets of preclinical cancer models. Nucleic Acids Res. 2019;47: D923–D929. doi:10.1093/nar/gky872

Walls AC, Park Y-J, Tortorici MA, Wall A, McGuire AT, Veesler D. Structure, Function, and Antigenicity of the SARS-CoV-2 Spike Glycoprotein. Cell. 2020;181: 281-292.e6. doi:10.1016/j.cell.2020.02.058

Wang K, Chen W, Zhang Z, Deng Y, Lian J-Q, Du P, et al. CD147-spike protein is a novel route for SARS-CoV-2 infection to host cells. Signal Transduct Target Ther. 2020;5: 283. doi:10.1038/s41392-020-00426-x

Wang S, Qiu Z, Hou Y, Deng X, Xu W, Zheng T, et al. AXL is a candidate receptor for SARS-CoV-2 that promotes infection of pulmonary and bronchial epithelial cells. Cell Res. 2021;31: 126–140. doi:10.1038/s41422-020-00460-y

Wrapp D, Wang N, Corbett KS, Goldsmith JA, Hsieh C-L, Abiona O, et al. Cryo-EM structure of the 2019-nCoV spike in the prefusion conformation. Science. 2020;367: 1260–1263. doi:10.1126/science.abb2507

Yao H, Song Y, Chen Y, Wu N, Xu J, Sun C, et al. Molecular Architecture of the SARS-CoV-2 Virus. Cell. 2020;183: 730-738.e13. doi:10.1016/j.cell.2020.09.018

Zhao Y, Kuang M, Li J, Zhu L, Jia Z, Guo X, et al. SARS-CoV-2 spike protein interacts with and activates TLR41. Cell Res. 2021;31: 818–820. doi:10.1038/s41422-021-00495-9
