# Supplementary material for: LRRC15 mediates an accessory interaction with the SARS-CoV-2 spike protein
Source: PLoS Biol. 2023 Feb 3;21(2):e3001959. doi: 10.1371/journal.pbio.3001959 (PMC9897555; doi:10.1371/journal.pbio.3001959)
Supplement: Attachment — Submitted filename: LRRC15 SARScov2 - rebuttal document REVISED2.pdf [file pbio.3001959.s046.pdf]

PBIOLOGY-D-21-02640R2

Reviewer #3:

The authors have performed amazing amount of work to answer my previous concerns. I greatly appreciate their extensive efforts and was really impressed by their careful dissection of these comments. The major issue now is the LRRC15 biology upon infection. Given the fact that two other papers suggested LRRC15 is inhibitory to SARS-CoV-2 in trans, it would be hard to evaluate the current data from this paper that LRRC15 modestly promotes infection in cis with ACE2. This observation is also not physiological as very little ACE2+ cells have LRRC15 expression. Nevertheless, this paper is still rather helpful to the research community. In this regard, it would be helpful to include into the paper the negative data from the authors that LRRC15 in trans could not inhibit SARS-CoV-2 real virus infection through ACE2, and potentially the validation data of other Spike receptors, Figure R5, which will raise important discussions to the field.

We are grateful to the reviewer for their kind words and valuable suggestions in improving our manuscript to the point it is, in their view, suitable for publication. We concur that the question about the physiological mechanism of LRRC15 during infection is a key one to resolve, and our latest round of revisions adds further useful data to provide exactly the kind of negative data the reviewer wisely notes is "helpful to the research community" and "raise[s] important discussions to the field".

Reviewer #2:

The authors have made appropriate revisions in response to input from the reviewers. Of note, the authors provided very extensive responses to every one of the reviewers' concerns, and they performed additional experiments. While not all experiments yielded useful findings, some did and they have been incorporated into the current version. Also notably, there are now three independent discoveries of LRRC15 as a SARS-CoV-2 binding ligand, and one report links LRRC15 to COVID19 outcomes. The findings in this submission mostly cohere with these other reports, and in those cases where they do not, the authors have offered some explanations for the perceived discordances. All in all, the authors communicated their findings clearly and professionally. Given that several groups have landed on LRRC15 as a SARS-CoV-2 binding ligand of potential significance to virus biology and COVID19, this paper is considered timely and highly significant to the field.

We thank the reviewer for their valuable input in improving our initial submission to reach the point that it is, in their view, suitable for publication. We fully agree that it is

now important that these results are published so that the community has access to all the data, particularly in cases where our results provide a counterpoint to claims made by others or raise but do not yet fully answer significant new questions for the field.

Reviewer #1:
the authors have not addressed my important concerns. It is important to analyse the co-expression of LRRC15 and ACE2. The addition of data on lung fibroblasts during inflammation is interesting but does not address the concern that LRCC15 cannot affect ACE2 positive cells or enhance infection.

major concerns
1. no data are provided that SARS-CoV-2 virus binds to LRRC15 (my initial question). The authors refer to other preprints but that is insufficient proof.
2. no data are provided that S binds recombinant LRCC15. there is a discussion about why it does not work and referred to a preprint, but this is not sufficient. the authors should show that S protein binds LRCC15.
3. no data are provided on the expression of S protein in more relevant cell-lines. some attempts have been made but no results have been obtained.
4. no primary virus is used and this decreases the relevance and impact.
5. trans infection. the data presented in R10 are not clear and do not really allow assessment of trans. a easier setup would be to incubate LRRC15 cells with SARS-CoV-2, wash after incubation period and add to ACE2 positive cells. this allows assessment of transmission and is unaffected by co-culture of cells. The experiments have not been performed well and therefore this needs to be revisited. the preprints showing transmission have used different more successful setup.

We thank the reviewer for their time in suggesting these further improvements for our revised manuscript. In this new second round of revisions, we have performed the experiment suggested by the reviewer to re-test the hypothesis that LRRC15 could modulate infection in *trans* by pre-incubation assays that would complement our earlier co-culture assay. Following the experimental setup recommended by the reviewer, we incubated our cell lines that express LRRC15 with wild-type SARS-CoV-2, then transferred the virions to susceptible ACE2-expressing target cells (Figure S9A). These data are provided in our revised manuscript as the new **Supplementary Figure 9**. As we had done in previous infection experiments, we took care to validate our assay using a positive control consisting of the known viral receptor ACE2, and ensured our clonal cell line expressing LRRC15 could express at high levels under an inducible promoter, thereby enabling tightly-controlled experiments where the exact same cell line in an uninduced state is used the reference comparison (Figure S9B).

For thoroughness, we tested a range of viral titers and for the cumulative effects of multiple rounds of viral passaging over the adsorbing cell type. Across all of these experimental configurations, we consistently detected no *trans* inhibition of infection (Figure S9C, Figure S9D).

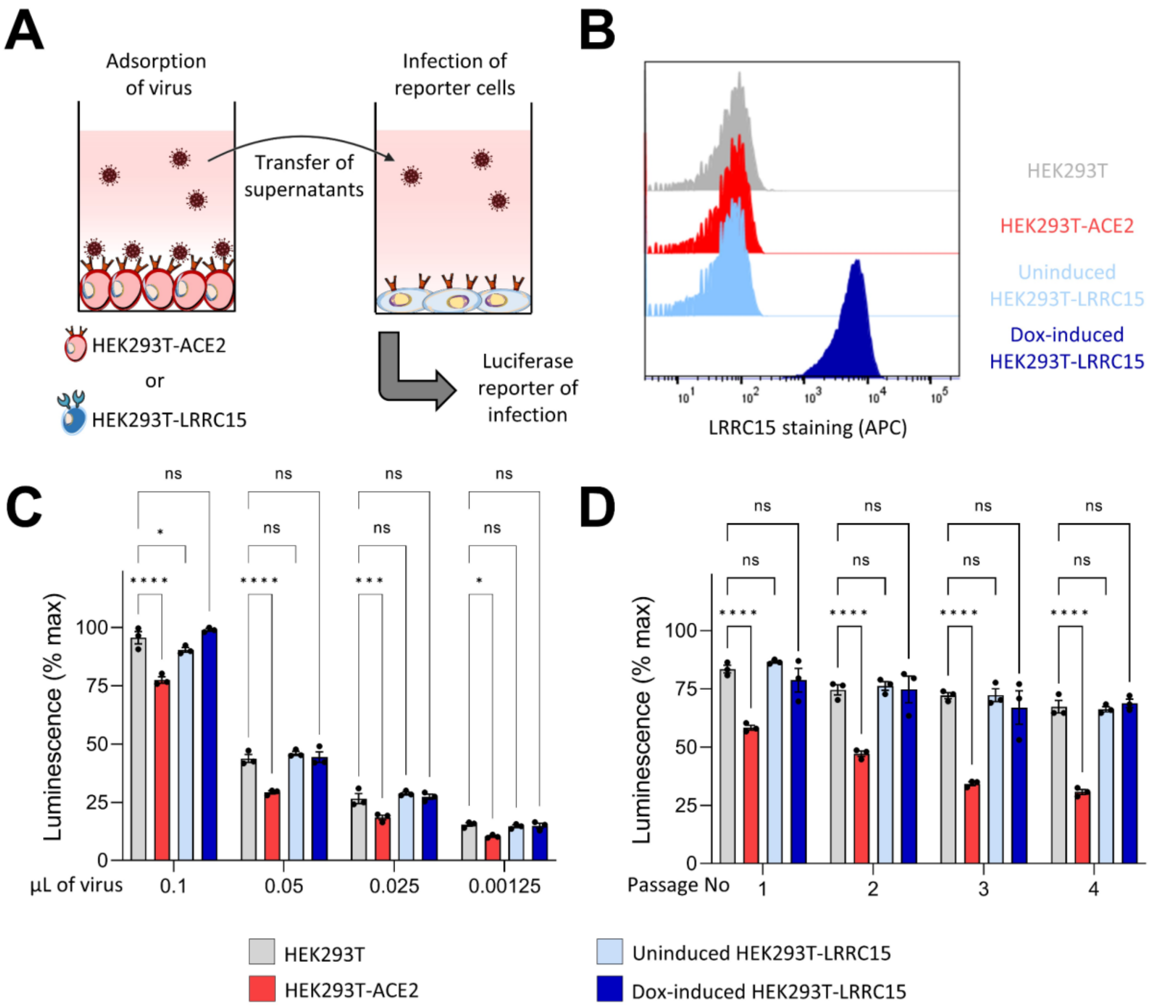

New Supplementary Figure 9. Testing for inhibition of SARS-CoV-2 infection in *trans* by LRRC15 using a viral adsorption assay finds no evidence for an inhibitory effect.

A. Schematic of the experiments. HEK293T cells express ACE2 or doxycycline-inducible LRRC15. Reporter cells express ACE2 and a luciferase-based biosensor of SARS-CoV-2 infection.

B. Surface LRRC15 expression can be readily induced by doxycycline with minimal background staining in the uninduced state. Flow cytometry traces show HEK293T cells in different expression conditions (red for constitutive ACE2 expression, light blue for uninduced LRRC15, blue for induced LRRC15 expression, and grey for unmodified HEK293T cells). Representative data from at least 4 independent experiments. These data are also referenced in Supplementary Figure 8B.

C. Pre-adsorption of authentic SARS-CoV-2 virus by HEK293T cells expressing ACE2 but not LRRC15 significantly reduces rates of infection across a range of MOIs. HEK293T, HEK293T-ACE2, uninduced HEK293T-LRRC15 or Dox-induced HEK293T-LRRC15 cells were

incubated with the indicated volume of SARS-CoV-2 viral stock for 4h to allow adsorption of viral particles. 0.1 µL of viral stock corresponds to MOI≈0.02. Supernatants were then transferred to reporter cells expressing ACE2 and a luciferase-based biosensor of SARS-CoV-2 infection. Infection of reporter cells is quantified as % maximum luminescence at 24h. Mean values ± SEM are shown for an experiment performed in triplicate, representative of 4 independent experiments.
D. Multiple rounds of pre-adsorption of authentic SARS-CoV-2 virus by HEK293T cells expressing ACE2 but not LRRC15 enhances the reduction in infection. HEK293T, HEK293T-ACE2, uninduced HEK293T-LRRC15 or Dox-induced HEK293T-LRRC15 were incubated with SARS-CoV-2 viral stock (MOI = 0.01) for 1.5h to allow viral adsorption (passage 1). A sample of each supernatant from passage 1 was stored at 4°C, and the remainder subject to a further round of pre-adsorption for 1.5h (passage 2). This step was repeated twice (total 4 passages). All supernatants were then transferred to reporter cells expressing ACE2 and a luciferase-based biosensor of SARS-CoV-2 infection. Infection of reporter cells is quantified as % maximum luminescence at 24h. Mean values ± SEM are shown for an experiment performed in triplicate, representative of 2 independent experiments.
Statistical significance was tested using two-way ANOVA followed by Dunnett's test for multiple comparisons. * $p < 0.05$, *** $p < 0.001$, **** $p < 0.0001$.

We have included these experiments suggested by the reviewer in our revised manuscript and they are significant in several respects. The first of these relates to one of the preprints that we referred to in our previous round of revisions as one of the studies which appeared after our manuscript's submission that replicated most of our findings about LRRC15 (Song *et al*, 2022 ; PMID: 36228039). Recently, PLOS Biology has published the work by Song et al. Despite our studies agreeing on most of the basic discoveries about LRRC15, one major point of divergence is that they claim (as the title of their manuscript and its central result) that LRRC15 inhibits infection in *trans*. However, the published data from Song *et al* do not include data for this claim using authentic (non-pseudotyped) virus, nor the combination of pre-incubation and co-culturing experiments we conducted. Crucially, our detailed new data provide that missing result, and it has profoundly changed the interpretation of the now-published claims made by Song *et al* in this same journal (PLOS Biology). Therefore, as emphasized by our other two peer reviewers when they praised the work we performed and recommended it be published, the negative results we provide are vital for the community to have in order to provide a needed counterpoint of how more physiological systems using wild-type virus contradict results that are now in the published record relying on pseudotype assays.

The extensive work we have performed to test the *trans* inhibition hypothesis is also valuable in how it points to our earlier finding of a *cis* effect of LRRC15 on modulating infection as the predominant functional mechanism. While we agree that not all of the details of how this *cis* effect occurs within the lungs of human patients are fully resolved (nor possible to fully resolve such a complex question with current methodologies and within the scope of the *Short Report* article we have submitted), the data we collected

testing alternative hypotheses have led to the conclusion that none of the other alternatives appear likely. Notably, on this point our results and those of Song *et al* are in generally good agreement. They likewise found that expression of LRRC15 in *cis* to ACE2 can have an enhancing function on the virus.

Given the intense interest our discovery of this LRRC15 interaction has attracted in the time since we first posted our pre-print over a year ago, we believe it is vital to present these data so that the virology community can have a more informed debate over the role of LRRC15 in infection and COVID-19. We appreciate the reviewers' input in helping to reveal these additional details of the infection mechanism which support the data we provided in our previous round of revisions.

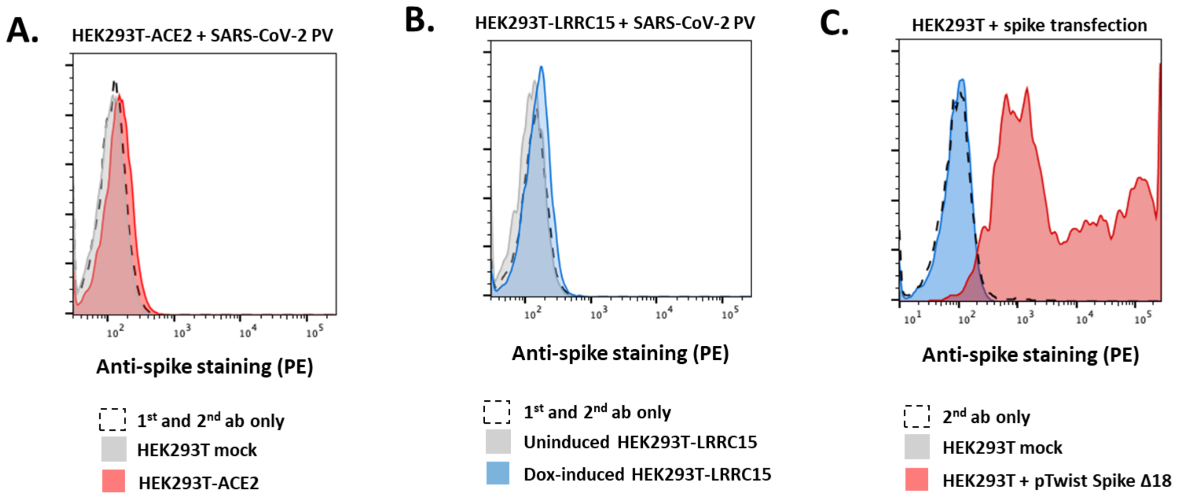

Figure R11. Binding of SARS-CoV-2 pseudoviral particles to HEK293T cells expressing ACE2 or LRRC15.

A-B. HEK293T, HEK293T-ACE2, uninduced HEK293T-LRRC15 or Dox-induced (250 ng/µL for 48h) HEK293T-LRRC15 cells were dissociated with Accutase, washed with ice-cold blocking buffer (PBS with 2% FCS) and incubated with SARS-CoV-2 spike-pseudotyped lentiviral particles (MOI≈0.5) for 45 min. Cells were then washed, stained with rat anti-spike (S1) antibody (Biolegend, cat. No. 944703, 2 µg/mL) for 30 min, washed and stained with a PE-conjugated anti-rat secondary antibody for 30 min. Finally, cells were stained with DAPI for 5 min, washed and analyzed by flow cytometry. Histograms show live (FSC/SSC-gated, DAPI-negative) cell populations. SARS-CoV-2 spike-pseudotyped lentiviral particles were generated according to standard methods by co-transfection of wild-type SARS-CoV-2 spike (pTwist Spike Δ18), Gagpol (pCMVΔR8.91) and a lentiviral transfer vector encoding GFP (pHRSIN-Ub-Emerald-PGK-Puro). To determine MOI, lentiviral stock was titrated by flow cytometry for GFP in HEK293T-ACE2 cells.

C. As a positive control, HEK293T cells were transfected with wild-type SARS-CoV-2 spike (pTwist Spike Δ18), harvested after 48 h, stained with rat anti-spike (S1) antibody and analyzed by flow cytometry as described above.

Next, with respect to comments about the interaction itself between LRRC15 and the spike protein of SARS-CoV-2, there likewise our study provides valuable context missing from the current literature. We would like to emphasize again that our manuscript explicitly states that it is not yet possible to conclusively rule-out the possibility that the interaction with LRRC15 is indirect as opposed to direct. As the reviewer correctly points out, because of the technical limitations we noted where the ectodomain of LRRC15 does not appear to be compatible with expression as a soluble recombinant protein that retains activity (also see similar conclusions reached by Cao *et al* 2021 ; DOI : 10.1073/pnas.2025451118), it is possible the interaction LRRC15 mediates might be indirect. Our manuscript is the only one to acknowledge this possibility, and provides a valuable counter-balance to claims made by Song *et al* which, as we discuss at length in our previous round of revisions (Figure R2), appear to be entirely predicated on the use of a faulty commercially-supplied recombinant LRRC15-Fc fusion protein reagent which lacks binding specificity. In our latest round of revisions, we did perform the experiment the reviewer suggested of trying to measure the direct attachment of virions to LRRC15-expressing cells, but through comparison to control cells expressing the much higher-affinity ACE2 receptor, we found that this assay lacked sufficient sensitivity to detect direct virion attachment even to ACE2 (Figure R11). Our revised manuscript includes additional text in the discussion to make this possibility of an indirect interaction more clear, given the significant implications this possibility would have on the burgeoning field of LRRC15:coronavirus research.

Lastly, we would like to respectfully point out that point #4 raised by the reviewer is factually incorrect. Primary virus experiments have been a key feature of our manuscript, from our very first submission when we used it in our BSL3 infection assays to discover LRRC15's modulatory effect on infection rates, and then again in our revision, we repeatedly used wild-type isolates of primary virus to test the hypothesis that LRRC15 could influence infection in *trans*. During this second round of revisions, yet again we have exclusively used primary virus in our infection assays. We have taken these great lengths to use infection by authentic isolates of SARS-CoV-2 because we agree with the reviewer how crucial it is to use primary virus to generate relevant and impactful data. Notably, we have used wild-type virus in these more technically challenging BSL3 experiments in order to provide much-needed data as a counterpoint to the recent results published by Song *et al* in PLOS Biology, which neglected to consider primary virus in several key experiments. Our results using primary virus have revealed a different conclusion, which has important implications for our understanding of this unique interaction between LRRC15 and the viral spike protein.
